# CFD-DEM-based simulation and performance analysis of key parameters in pneumatic high- speed precision maize seed-metering device

**DOI:** 10.3389/fpls.2025.1700037

**Published:** 2025-10-28

**Authors:** Junzhi Chen, Shaobo Qi, Fudong Xu, Pengcheng Jia, Zixin Yuan, Dejun Xi, Hongyang Xu, Jinwu Wang

**Affiliations:** ^1^ College of Engineering, Northeast Agricultural University, Harbin, China; ^2^ Heilongjiang Provincial Scientific Research Institute of Agricultural Machinery Engineering, Suihua, China

**Keywords:** precision seeding, aperture, inner diameter of seed guide tube, airflow-assisted transport, bench test

## Abstract

To reduce seed loss, miss and multiple seeding in high-speed precision sowing, a pneumatic high-speed precision maize seed metering device was designed and analyzed in this study. Single-factor CFD simulations were conducted for the diameter of the seedling tray shaped holes and the inner diameter of the seed guide tube, respectively. Results showed that a hole diameter of 4.5 mm offered the best balance between suction efficiency and stability at different angular positions on the seed plate. Likewise, a seed guide tube inner diameter of 22 mm resulted in more uniform airflow and more consistent seed acceleration. A subsequent coupled CFD-DEM simulation was conducted, beginning with single-factor studies to identify a negative pressure range of -4 to -6 kPa that ensures reliable seed pickup and transport. Multi-factor simulations then incorporated positive pressure assistance in the seed guide tube. Bench tests at a forward velocity of 10 km/h confirmed that the best parameter combination was a negative pressure of -4 kPa and a positive pressure of 3 kPa, achieving a seed qualification rate of 96.48% and a coefficient of variation of 10.90%. The relative error between simulation and experimental results was within 5%, demonstrating excellent agreement and effective seeding uniformity.

## 1 Introduction

Maize, as one of the world’s most essential staple crops, plays a vital role in food security, provides livestock feed, and supports bioenergy production (Di Salvo et al., 2021; Wang et al., 2021b, Wang et al., 2021a; Zhang et al., 2023). With the rapid advancement of modern agricultural mechanization, the effectiveness of precision sowing has become a key factor influencing seed germination, crop establishment, and final yield (Shun et al., 2020; Tang et al., 2023a). Therefore, developing a high-efficiency seed metering system capable of maintaining sowing accuracy at high-speeds is crucial for improving maize production efficiency and ensuring planting quality.

Seed metering devices are generally categorized into two types: mechanical and pneumatic, based on their operating principles. Pneumatic precision seed metering devices are gradually replacing traditional mechanical seed metering devices due to their good adaptability to seed morphology and stable single-seed metering performance under high-speed conditions. Mechanical seed metering devices are limited by their rigid structure and insufficient response speed, and often suffer from problems such as seed injury, miss seeding and multiple seeding during high-speed operations, thus limiting their application in large-scale mechanized agricultural production (Yu et al., 2011; Liao et al., 2012; Wang et al., 2017; Zhou et al., 2018; Wu et al., 2020). On the other hand, pneumatic seed metering devices use negative pressure to pick up seeds and positive pressure to release them, providing several benefits such as simple design, good adaptability to different seed shapes, and more consistent sowing (Liu et al., 2011; Yatskul and Lemiere, 2018; Mengjie et al., 2021; Hu et al., 2021). Currently, pneumatic seed metering systems are being increasingly used for planting crops such as maize, soybeans, and rapeseed, demonstrating excellent flexibility and reliable performance in various field conditions (Li et al., 2025). In recent years, scholars at home and abroad have proposed a variety of structural innovations for pneumatic seed metering devices, including internally filled seed trays, dual-chamber structures, and airflow-assisted seed guiding (Liu et al., 2022; Ding et al., 2024; Sun et al., 2024). These studies have made significant progress in understanding the mechanism of airflow-seed interaction, while multiphysics field coupling methods (e.g., CFD-DEM) have been widely used to analyze gas-solid coupling and the laws governing particle microscopic motion (Li et al., 2024; Han et al., 2025; Wang et al., 2025). However, existing results are still primarily focused on low and medium-speed conditions, and insufficient attention has been paid to the flow field characteristics, seed movement, and seed discharge consistency under high-speed conditions (Guo et al., 2024; Ding et al., 2025). In addition, although some studies have performed single-factor optimization of the diameter of seed suction holes, airflow velocity, or seed guide tube structure, there is a lack of systematic analyses that combine structural improvement with synergistic optimization of airflow (Zhang et al., 2023; Xing et al., 2024). This shortcoming underscores the urgent need for a joint study on the structural design and pneumatic regulation of seed metering devices to ensure reliable single-grain adsorption and uniform spacing under high-speed seeding conditions.

In the pneumatic seeding system, key parameters such as the size and distribution of shaped holes in the seed tray, along with the structural features of the inner diameter of the seed guide tube, directly influence the force and movement of seeds during seeding. A well-designed configuration of these components is essential for ensuring accurate and stable sowing at high operational speeds. In addition to optimizing seed tray structures, existing research has increasingly emphasized the role of airflow-assisted seed guiding structures in pneumatic precision seed metering systems. The results of bench tests on internally inflated and blown precision seed metering devices showed that, although such devices have a high pass rate, the coefficient of variation of plant spacing is significant, and the issue of seeding uniformity remains prominent (Cui et al., 2017). To address this shortcoming, related researchers have all proposed seed guide tube designs with airflow assistance to stabilize the seed trajectory. Yu et al. (2021) investigated the key structural parameters of the curved seed guide tube by using a coupled CFD-DEM method, and concluded that if the seed guide tube is inclined and curved, the conical structure with a wide top and narrow bottom can better constrain the seed drop; Wang et al. (2023) utilized a positive-pressure airflow jet during seed drop to accelerate the seed drop and reduce the deviation brought by the free-fall. The Dutch company Lockwood Inc. (Lockwood, the Netherlands) developed a potato air-aspirated seed metering device that utilizes positive pressure airflow to convey throw seed to improve seed drop consistency under high-density conditions (Li et al., 2020). However, because of the complexity of the gas-solid coupling mechanisms involved, it is difficult to precisely describe seed motion during sowing using only empirical methods or simplified mechanical models. This highlights the importance of advanced simulation techniques in systematically studying the multiphysics interactions among seeds, airflow, and structural components. Computational Fluid Dynamics (CFD) combined with the Discrete Element Method (DEM) has become a valuable tool for analyzing the microscale behavior of granular materials in complex airflow environments (Sun et al., 2025). Most current studies mainly focus on medium and low-speed operations, despite the increasing use of CFD-DEM modeling to optimize pneumatic seed delivery systems. There is still a lack of detailed research into flow field dynamics, seed movement, and distribution consistency under high-speed sowing conditions (Xing et al., 2024). Li et al. (2020) analyzed the relationship between different lengths of seed guide tubes and airflow distribution. They found that seed guide tube geometry directly affects the uniformity of the airflow field and the location of seed drops, while Yazgi (2016) showed that different seed guide tube types significantly impact the uniformity of plant spacing, demonstrating the sensitivity of the seed metering system to structural changes. In summary, the combination of airflow-assisted seed guide structure and pneumatic seeding system has essential research significance. High-speed precision seed metering devices not only need to optimize the design of the seed suction hole, but also must rely on the synergistic effect of the airflow-assisted seed guide.

Based on the considerations mentioned above, this study introduces a pneumatic high-speed precision seed metering device specifically created for maize. A CFD-DEM coupled simulation approach was used to examine the essential structural parameters and the maize seed transport mechanism. By comparing the simulation results with bench-scale experimental data, the viability and efficiency of the improved structural design and operational settings were confirmed. The results of this study offer both a theoretical basis and practical engineering guidance for optimizing the structure and parameters of pneumatic high-speed precision seed metering systems.

## 2 Materials and methods

### 2.1 Overall structure and working principle of pneumatic high-speed maize precision seeding device

**Figure 1:**
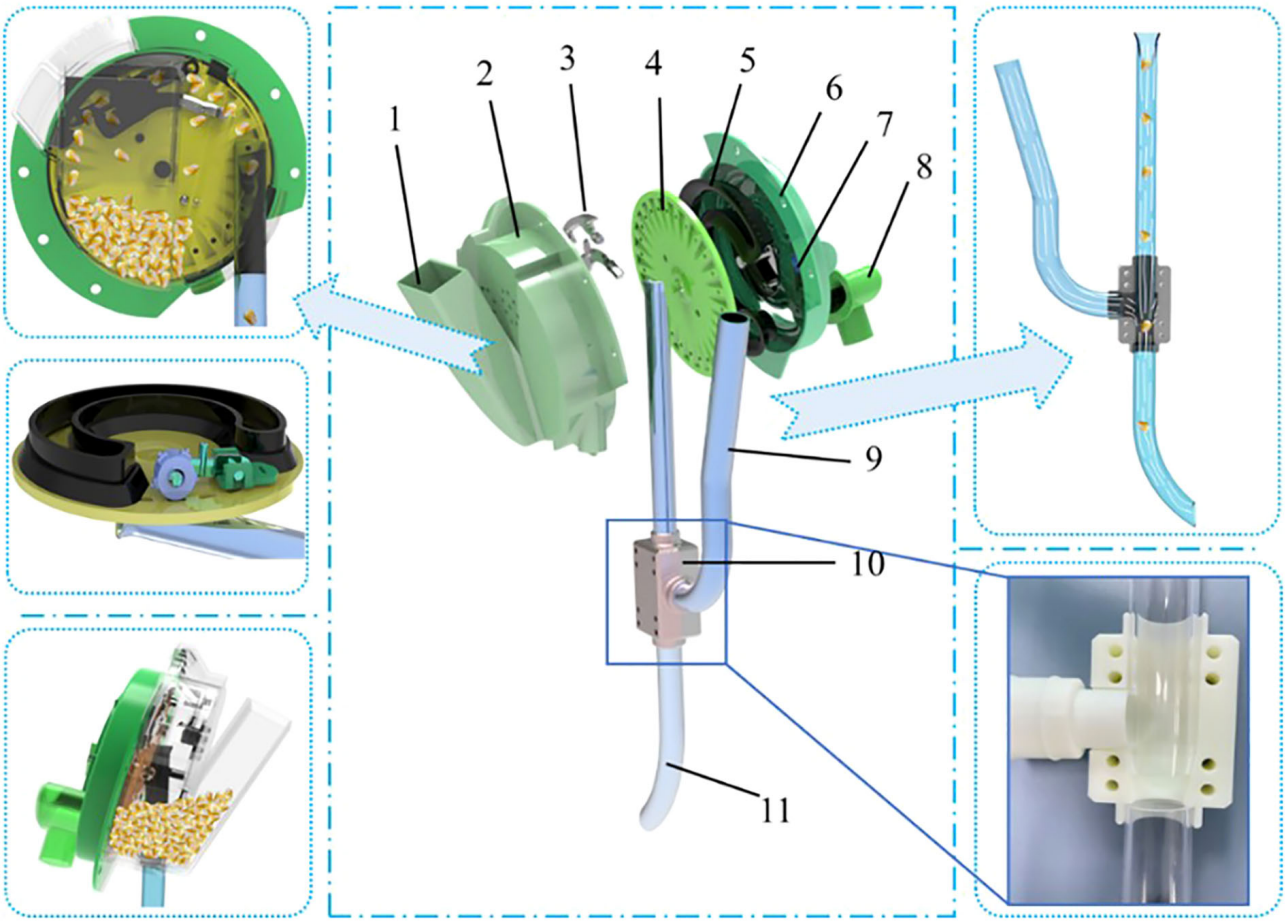
Overall structure of the pneumatic high-speed precision maize seed metering device. Overall structure of the pneumatic high-speed precision maize seed metering device. 1. entry point 2. front shell 3. seed-cleaning knife 4. seed tray 5. negative pressure chamber 6. rear housing 7. seed pressing wheel 8. air intake 9. positive pressure air inlet 10. airflow control valve 11. throw-seed tube.

The pneumatic high-speed maize precision seed metering device has several key parts, including the front housing, seed-cleaning knife, rear housing, inclined seed tray, negative pressure chamber, seed pressing wheel, and air-assisted seed dropping device. The airflow-assisted seed guide assembly consists of the seed guide tube, positive pressure air inlet tube, and an airflow directional valve. The negative pressure chamber and seed pressing wheel are mounted on the rear housing, while the seed-cleaning knife is attached to the front housing, which also has a seed inlet and a seed positioning port. The seed guide tube connects to the positioning port on the front housing. The airflow-assisted device is divided into two functional zones: a seed acceleration zone and a seed release zone. Additionally, the rear housing features a negative pressure air inlet that connects directly to the negative pressure chamber, as shown in the accompanying **Figure 1**.

During operation, the negative pressure fan creates a steady airflow through the negative pressure inlet, drawing maize seeds into the seed filling zone via the seed inlet. The drive motor then starts rotating the inclined seed tray, beginning the seed metering process. As the shaped hole on the disc approaches the filling zone, the airflow and the grooves around the shaped holes agitate the seeds, encouraging seed pickup. A seed-cleaning knife then removes excess or misaligned seeds, which fall back into the filling area. The rotating disc transports the remaining seeds that are securely attached to the seed-dropping zone. When exiting the negative pressure chamber, the seeds enter the seed guide tube under the combined influence of gravity and centrifugal force, completing the seed release process. To prevent blockages, a seed discharge wheel actively clears any seeds that fail to enter the seed guide tube smoothly. Finally, the seeds fall accurately into the soil with the assistance of airflow, completing high-speed precision sowing.

The structure and workflow reveal that the key parts affecting seed quality are the seed tray and the seed guide tube. The first manages how effectively seeds are picked up by negative pressure airflow, while the second influences the speed and direction of seeds as they fall. Achieving optimal seeding results depends on the coordinated operation of these two components, ensuring both accuracy and stability at high-speeds. The upcoming sections will analyze the seed tray and seed guide tube to understand their functions and contributions to overall seeding performance.

### 2.2 Mechanism analysis of seeds in seed trays and airflow-assisted seed delivery devices

#### 2.2.1 Analysis of seed transport mechanisms during seed dispersal and seed carrying processes

The seed tray is a key part of a high-speed precision seed metering device and is crucial for ensuring good seeding quality (Yuhuan et al., 2019; Li et al., 2019; Qinghui et al., 2020; Lai et al., 2020). In this study, the diameter of the seed tray was set to 166 mm, based on the team’s previous research findings (Jia et al., 2018). The disc has 26 seed suction holes evenly spaced around its edge (Liu et al., 2010). The negative pressure zone covers about three-quarters of the disc’s circumference, including roughly 19 to 20 of the seed suction holes.

Under stable negative pressure airflow conditions, the airflow velocity and pressure within each shaped hole are mainly affected by factors such as the diameter of the seed suction holes, the angular position of the shaped hole on the disc, and the aerodynamic interference between neighboring holes. As shown in Equation 1, the adhesive force exerted on the seeds at each shaped hole can be described as:


(1) $$F_{i}=\Delta P_{i}\cdot A_{i}=(P_{\text{atm}}-P_{i})\cdot \frac{\pi D_{i}^{2}}{4}$$

where *P_i_* represents the local pressure within the *i*-th suction shaped hole, and *D_i_* stands for the corresponding hole diameter. Typically, stable seed adsorption occurs when the adhesive force *Fi* exceeds the combined effects of gravitational force, frictional resistance, and centrifugal force acting on the seed. In porous systems, factors such as mutual interference between pore channels, the constantly changing spatial relationship between shaped holes and the position of the negative pressure inlet, and the dynamic entry and exit of pores into and out of the negative pressure zone during turntable rotation lead to non-uniform pressure and airflow velocity distributions across the pores. These variations markedly influence the stability of seed adsorption.

**Figure 2:**
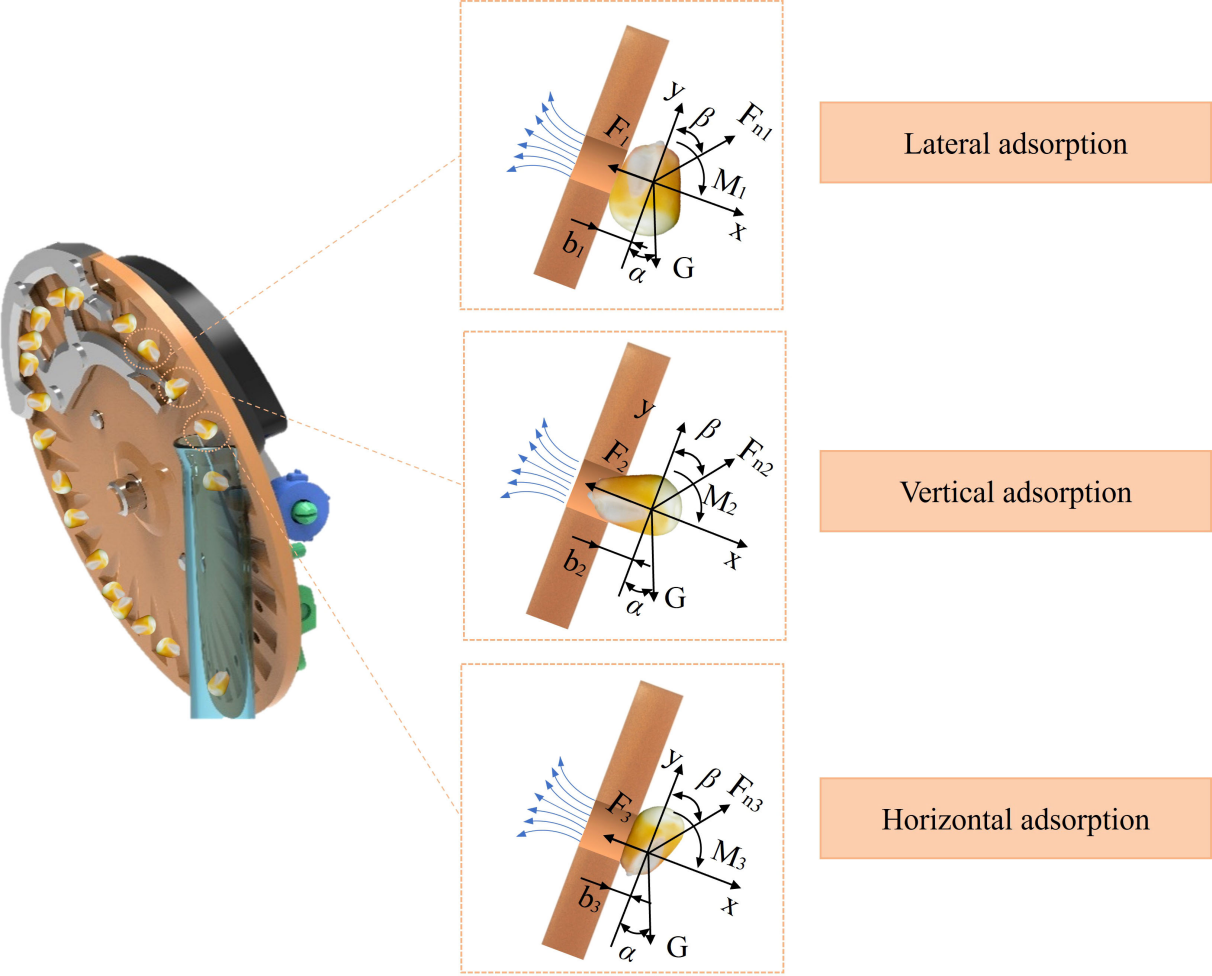
Seed adsorption posture analysis.

Therefore, an analysis was performed on the seeds absorbed within the shaped hole, as shown in **Figure 2**. Since the seeds were in a relatively stable state after excess seeds were removed by the seed-cleaning knife, friction between the seeds and the seed tray, as well as centrifugal forces and other minor effects, was ignored. Instead, the results were expressed in terms of torque and moment of inertia, which offered a more robust and convincing representation of the seed behavior.

Since the forces acting on the three adsorption postures are pretty similar, the equations are simplified to make it easier to analyze the key variables and draw meaningful conclusions. The analysis of the three seed adsorption postures is shown in Equation 2:


(2) $$\left\{ \begin{array}{l} G\cos\alpha -F_{n1}\cos\beta =ma_{yi}\\ F_{i}-F_{ni}\sin\beta =ma_{xi}\\ G\cos\alpha b_{i}+F_{ni}\cos\beta b_{i}=M_{i}=J_{\omega }\alpha_{i} \end{array}\right.$$

Simplify the formula to calculate *a_yi_* as shown in Equation 3.


(3) $$a_{yi}=\frac{G\cos\alpha -F_{ni}\cos\beta }{m}$$

Simplify the formula to calculate *α_i_* as shown in Equation 4.


(4) $$\alpha_{i}=\frac{b_{i}(G\cos\alpha +F_{ni}\cos\beta )}{J_{\omega }}$$

According to the simplified formula, *α_i_* represents the angular acceleration, which is mainly affected by the variable *b*. As shown in the force analysis diagram, when seeds are adsorbed in a horizontal position, the value of *b* is the smallest, indicating the lowest angular acceleration and, therefore, the most stable adsorption state. Similarly, the analysis of *a_yi_* showed no significant differences among the three adsorption postures. Consequently, it can be concluded that horizontal adsorption offers the most effective and stable seed attachment.

Additionally, the seed adsorption posture not only affects seed distribution performance but is also influenced by the diameter of the suction shaped holes. While previous studies have mainly focused on overall sowing performance, limited attention has been given to the specific effect of shaped hole diameter on seed adsorption efficiency. The diameter of the suction shaped holes has a dual effect on adsorption performance. On one hand, a larger hole diameter increases the cross-sectional area, which enhances the suction force under a constant pressure differential. On the other hand, overly large holes can decrease local pressure and cause increased airflow leakage, ultimately reducing the effective pressure differential. Conversely, if the hole diameter is too small, it may maintain a high-pressure differential but fail to ensure stable adsorption due to a limited contact area. In some cases, it can even produce high-velocity jets that disturb the surrounding airflow. Moreover, suction holes near the edges of the negative pressure zone are more prone to pressure and velocity fluctuations, making them high-risk areas for multiple seeding or miss seeding.

Therefore, to thoroughly study the seed discharge performance caused by changes in pressure and airflow velocity within the shaped hole under different hole diameter conditions—and to assess the stability of adsorption for each hole throughout the operating cycle—a comprehensive three-dimensional flow field simulation model is necessary. In this study, a CFD-based simulation method is used to systematically analyze the local pressure distribution and velocity field variations associated with different hole diameter configurations. The aim is to find the optimal hole diameter parameters that allow for more precise single-seed adsorption and dependable seed release, thereby improving the overall performance of the seed metering system.

#### 2.2.2 Analysis of the transport mechanism during seed transfer

**Figure 3:**
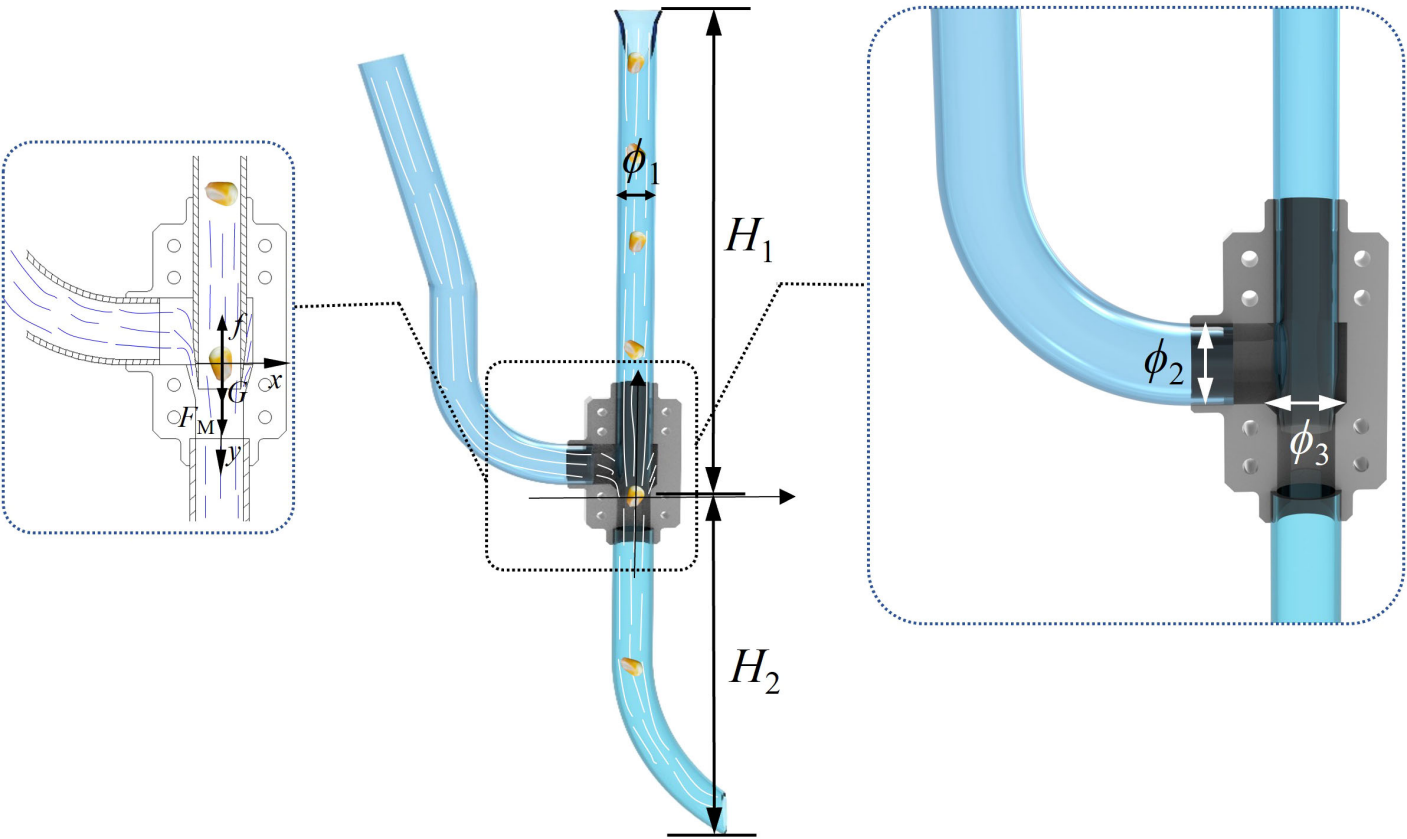
The structural dimensions of the fully constrained airflow-assisted seed guide device.

In previous studies, the influence of seed guide tubes on sowing quality in pneumatic maize precision seed metering device has been investigated (Tang et al., 2023b). In this study, a left-positioned seed guide tube is used, into which positive pressure airflow is introduced to assist the seed dropping process, as shown in **Figure 3**.

Fluid drag results from the relative motion between particles and the surrounding fluid. When the airflow velocity greatly exceeds the particles’ velocity, the drag force acts as a propulsive force, effectively pushing the particles forward (El-Emam et al., 2021). As shown in Equation 5:


(5) $$F_{M}=\frac{1}{2}C_{D}\rho Av_{△}^{2}$$

where *F*_M_ represents the seed drag force in the airflow, measured in N; *C_D_* is the drag coefficient; *ρ* is the fluid density in kg/m³; *A* is the maximum cross-sectional area of the particle in m²; and *v*_△_ is the relative velocity between the particle and the fluid in m/s.

Since the shape of maize seeds has little impact on speed, we will assume the seeds are spheres for analysis. For spherical bypasses, the drag coefficient *C_D_* can be determined as shown in Equation 6.


(6) $$C_{D}=\{\begin{array}{l} \frac{24}{R_{e}}(R_{e}⩽0.5)\\ \frac{24(1.0+0.15R_{e}^{0.687})}{R_{e}}(0.5<{\text{R}}_{e}<1000)\\ 0.44(R_{e}>1000) \end{array}$$

where *R_e_* is the Reynolds number, which is less than 2300 for laminar flow and greater than 2300 for turbulent flow. The Reynolds number is calculated as shown in Equation 7:


(7) $$\left\{ \begin{array}{l} R_{e}=\frac{\rho v_{d}d}{\mu_{0}}\\ s_{m}v_{m}=s_{d}v_{d} \end{array}\right.$$

where *ρ* is the fluid density, measured in kg/m^3^; *d* is the seed guide tube diameter in meters; *μ* is the viscosity, with units of Pa·s; *s_m_* is the cross-sectional area of the seed guide tube inlet, in square meters; *s_d_* is the cross-sectional area of the seed guide tube outlet, in square meters; *v_d_* is the gas flow velocity at the seed guide tube outlet, expressed in m/s.

Under standard conditions, the air density is 1.29 kg/m^3^, and the viscosity is 1.8 × 10–^5^ Pa·s. According to the built-in analysis software in the 3D modeling program, the cross-sectional area of the intake port is 0.000655 m^2^, and the diameter of the intake holes ranges from 0.004 m to 0.0055 m. The cross-sectional area of the seed-feeding tube’s inlet is 0.000176 m^2^, and the diameter of the seed-feeding tube’s outlet is 0.002 m. Based on the formula, the Reynolds number is well above 2300. Therefore, the air resistance coefficient *C_D_* for rapeseed in this study is 0.44.

Overall, positive pressure airflow is introduced into the throw-seed tube system through the left side pipeline. Due to the high airflow velocity, the seeds experience a downward drag force both within the seed guide tube and inside the throw-seed tube. As a result, a comprehensive force analysis is necessary to assess the motion of the seeds throughout the entire conveying pipeline. The seeds are subjected to the combined effects of aerodynamic thrust from the high-speed airflow and gravity, leading to continuous acceleration in the vertical direction. According to Newton’s second law, the seed’s acceleration equation can be written as shown in Equation 8.


(8) $$F_{M}+G=\frac{4}{3}\rho_{m}\pi r_{m}^{3}\frac{dv_{m}}{dt}\times 10^{-9}$$

where *F_M_* is the seed drag force in the airflow, with units of N; *G* is the seed’s gravitational force, with units of N; *r_m_* is the average maximum width of the test seeds, with units of m; *v_m_* is the velocity in the airflow, with units of m/s; and *ρ_m_* is the air density, with units of kg/m³.

Based on the kinematic and dynamic models of maize seeds in the seed delivery tube from the previous stage, along with the external dimensions of the seeds, the specific structural parameters of the airflow-assisted seed delivery device are designed as follows:

1. Seed guide tube diameter *ϕ*_1_: To ensure that maize seeds can smoothly enter the seed guide tube and be accelerated, the diameter of the seed inlet is related to the three-dimensional size of the maize seeds. The maximum dimension of the maize seeds was measured to be 12 mm, as detailed later in this article. Therefore, the diameter of the seed inlet should be slightly larger than the maximum size of the maize seeds. It was determined that the diameter of the seed inlet should be greater than 18 mm. The specific parameters were then established using a single-factor simulation method.
2. Seed guide tube height *H*_1_: To achieve low-position seeding, the seed tube should be set to a moderate height. Based on the overall dimensions of the no-till seeder, the seed tube height was determined to be 300 mm.
3. Throw-seed tube height *H*_2_: Since the throw-seed tube is located below the deflector valve, the airflow velocity inside the tube is much higher than that inside the seed guide tube. Therefore, to ensure that the maize seeds are sufficiently accelerated without becoming too fast, the height is set to 200 mm.
4. Inlet diameter *ϕ*_2_: The size of the intake port in the airflow-assisted seed guiding device directly influences the flow rate of positive pressure airflow entering the deflector valve. To ensure stable airflow, the intake port diameter is designed to be 20 mm.
5. The diameter of the deflector valve *ϕ*_3_: One end of the deflector valve connects to the air inlet, providing a stable downward positive pressure airflow to the seed guide tube. This helps the maize seeds inside the seed guide tube accelerate steadily. Therefore, the diameter of the deflector valve is crucial for preventing air from flowing back within the seed guide tube. Since the inner diameter of the seed guide tube should be greater than 18 mm to allow smooth airflow through the deflector valve into the throw-seed tube, the deflector valve is designed to be 34 mm.

### 2.3 Numerical simulation analysis of the seed sowing process

Throughout the entire seed-dispensing process, maize seeds experience a complex flow field environment. The diameter of the sowing planter’s shaped holes affects the effectiveness of airflow adsorption on the seeds, while the inner diameter of the airflow-assisted seed guide tube influences the velocity and stability of the airflow during seed descent. To evaluate sowing quality, single-factor CFD simulations were conducted to analyze the effects of sowing planter, shaped hole diameter, and seed guide tube inner diameter. Additionally, a coupled CFD-DEM simulation was used to examine the seed dispensing mechanism from a microscopic perspective, offering deeper insights into the interaction between airflow and seed particles.

#### 2.3.1 Analysis of the transport mechanism during seed transfer

(1) Simplification of the sowing planter and air-assisted seed sowing device model and mesh partitioning.

**Figure 4:**
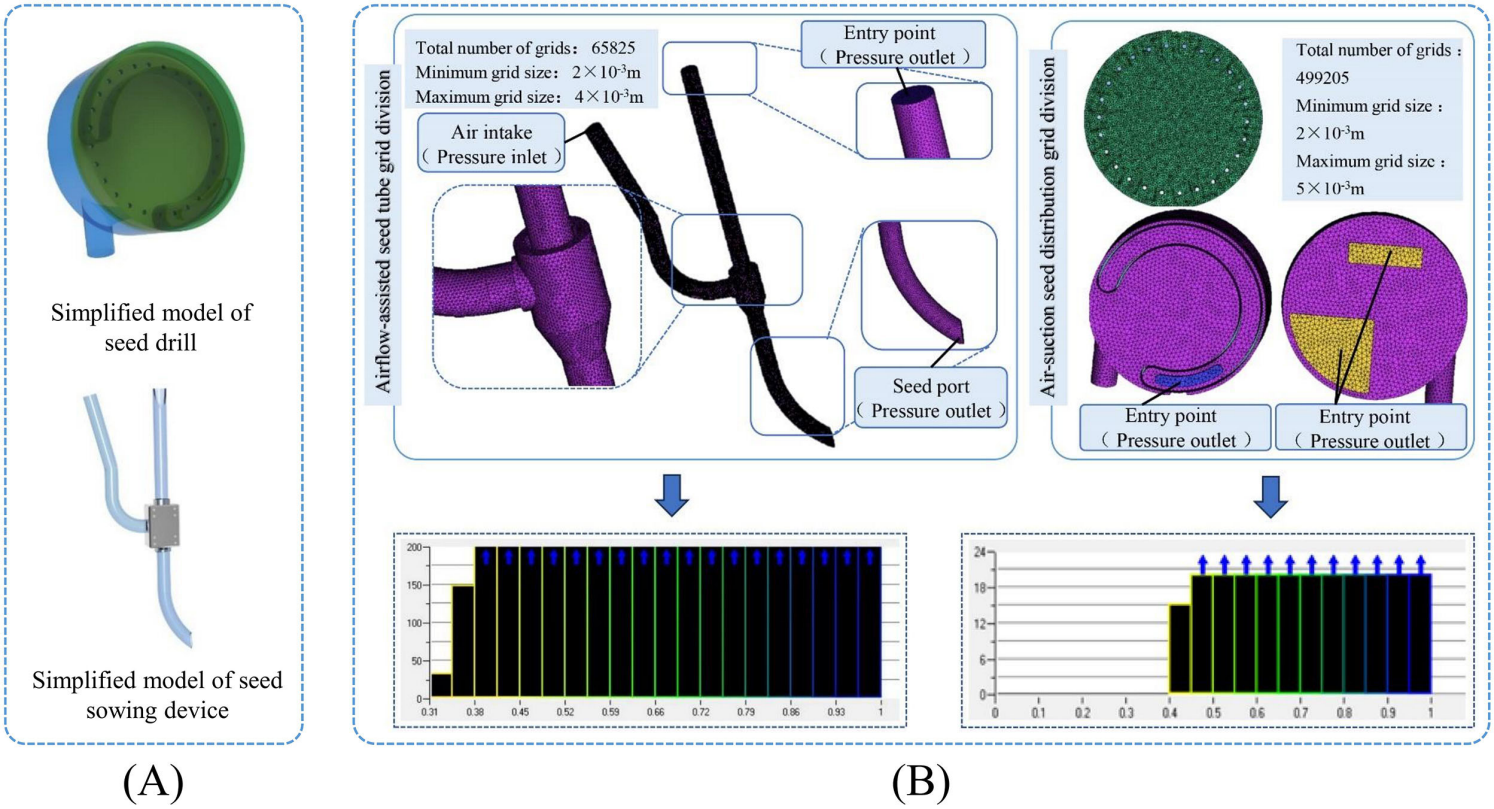
Model simplification and grid generation. **(A)** Simplified model of pneumatic high-speed precision maize seed metering device; **(B)** Grid generation and grid quality.

The three-dimensional model created in the initial study was simplified using SolidWorks (Dassault Systèmes Company, MA, USA). The seed metering assembly was kept with key components, including the seed tray, front and rear housings, negative pressure chamber, and seed-clearing blade. Additionally, the airflow-assisted seed delivery unit was retained to ensure the system functions properly. As shown in **Figure 4A**.

In ANSYS 22.0 (ANSYS, Pennsylvania, USA), the meshing module was used to discretize the simplified model described above. To improve the accuracy of numerical simulations, local mesh refinement was applied to the sowing planter shaped hole and the auxiliary seed delivery components. After completing the meshing process, the minimum element size was set to 2 × 10^-3^ m, the maximum element size to 5 × 10^-3^ m, and the overall mesh quality was kept above 0.3 to ensure reliable computational results. After the seed feeding device division, the minimum grid size is set to 2 × 10^-3^, the maximum grid size to 4 × 10^-3^, and the grid quality exceeds 0.4, all meeting the simulation requirements. As shown in **Figure 4B**.

(2) Establishment of a maize seed model.

**Figure 5:**
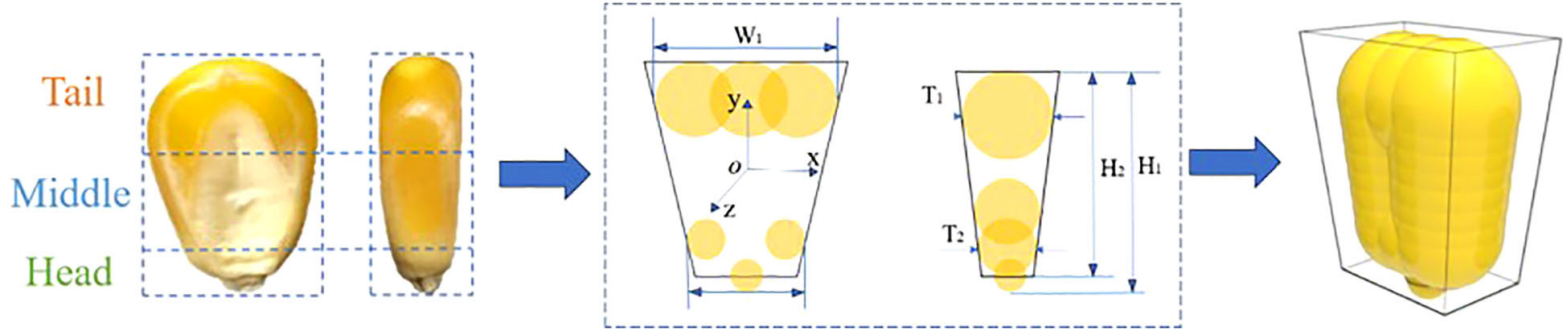
Establishment of a simulation model for maize seeds.

The simulation experiments selected “DeMeiya No. 1” maize seeds, which are widely cultivated in the northeast region, as the research object. These seeds are shaped like horse teeth. To improve measurement accuracy and representativeness, a vernier caliper was used to measure the surface structural parameters, with 100 seeds randomly selected for measurement. The average dimensions of their three axes were calculated using SPSS 26 software (SPSS, Chicago, IL, USA), resulting in mean and standard deviation values of (12.35 ± 0.33) mm, (9.24 ± 0.42) mm, and (4.70 ± 0.57) mm, respectively. To describe the external structural features of the seeds, the following parameters are defined: W_1_ as the width of the widest part of the seed tail, W_2_ as the width of the head, T_1_ as the tail thickness, T_2_ as the head thickness, H_1_ as the overall height, and H_2_ as the height after removing the tip. Based on the multi-sphere modeling method, “DeMeiya No. 1” maize seed particles were modeled in EDEM 2020 (Altair company, Michigan, USA). The seed head was filled with spheres of radius 1.3911 mm, while the middle and tail sections were filled with spheres of radius 2.2484 mm, as shown in **Figure 5**.

(3) Determination of simulation boundary conditions.

Since the simulation involves airflow fields, it is essential to determine these fields. Identifying the fundamental physical properties of a flow field is a necessary step before running the simulation. The compressibility of a fluid’s basic physical properties is categorized into laminar flow and turbulent flow. Whether a fluid is compressible depends on the Mach number (*M_a_*), with 0.3 serving as the critical threshold. Specifically, a fluid is considered compressible when *M_a_* > 0.3 and incompressible when *M_a_* < 0.3.

The Mach number formula is shown in Equation 9:


(9) $$M_{a}=\frac{v_{m}}{a_{m}}=\sqrt{\frac{v_{m}^{2}}{KRT}}$$

where *v_m_* is the velocity of the flow field in the negative pressure chamber, with units of m/s; *a_m_* is the speed of sound, with units of m/s; *K* is the ratio of specific heat at constant pressure to specific heat at constant volume; *R* is the gas constant, with units of J/(kg·K); *T* is the absolute temperature, with units of K. The choice between laminar flow and turbulent flow is determined based on the Reynolds number, as mentioned earlier.

The suction port of the negative pressure chamber in the pneumatic seed distribution system is set as the pressure inlet with a pressure of -4.5 kPa. The seed inlet, seed guide tube inlet, and air inlet are designated as pressure outlets, each with an outlet pressure of 0 kPa. In Fluent settings, the inlet of the under-constrained airflow-assisted seed guide tube is configured as a pressure inlet with a pressure of 3.0 kPa. The seed discharge port and seed inlet of the seed guide tube are set as pressure outlets with a pressure of 0 kPa. The near-wall surface uses the standard wall equation. For simulating a single-factor airflow field, a pressure-based solver is used, which can correct both the momentum and pressure of the flow field and couple pressure with velocity.

Based on the results of single-factor flow field simulation, additional simulation tests were performed using the CFD-DEM coupling method to analyze the filling behavior of maize-coated seeds in a pneumatic high-speed precision maize seed metering device and the seeding efficiency of an under-constrained airflow-assisted seed guide tube. The Eulerian coupling model was chosen for the simulation calculations. The Hertz-Mindlin no-slip contact model was used in EDEM, while the simplified coupling model was imported into Fluent. The specific simulation parameters are shown in **Table 1**. The CFD-DEM coupling interface used the Euler coupling type, with the annotated *k-ϵ* turbulence model, the Ergun and Wen & Yu models for drag, and other models, such as lift and heat transfer, set to default values. During coupling, the Fluent time step is typically 50–100 times larger than the EDEM time step. The EDEM and Fluent time steps were set to 2.0 × 10^−6^ s and 1.0 × 10^−4^ s, respectively. The total simulation time was set to 5 s, with experimental data saved every 0.01 s.

**Table 1:** Coupled simulation parameters.

Phase state	Parameters	Maize seed	Acrylic	Reference
Solid-state	Poisson ratio	0.40	0.50	(Wang et al., 2017; Jinwu et al., 2017)
	shear modulus (Pa)	1.37×10^6^	1.77×10^8^	(Kruggel-Emden and Oschmann, 2014)
	density (kg/m^3^)	1197	118	
	restoration coefficient	0.182	0.709	(Wang et al., 2016)
	static friction coefficient	0.0338	0.4590	
	coefficient of rolling friction	0.0021	0.00931	(Shi et al., 2020)
Gaseous	fluid	air		
	gravitational acceleration (m/s^2^)	9.81		(Kruggel-Emden and Oschmann, 2014)
	density (kg/m^3^)	1.225		
	viscosity (kg/m/s)	1.7984×10^-5^		

#### 2.3.2 Simulation test content and methods

##### 2.3.2.1 Single-factor simulation test factors and indicators

In the analysis of the flow field in the simulation test, the diameter of the shaped hole in the seed tray is the test factor. In contrast, the air pressure and air velocity inside the hole are the test indicators. The negative pressure of the sowing planter was set to 4.5 kPa, meaning the air pressure at the inlet was 4.5 kPa and at the outlet was 0 kPa. Considering that the shaped hole diameter range of the seed tray was 4-5.5 mm, the diameters were set to 4 mm, 4.5 mm, 5 mm, and 5.5 mm to conduct single-factor experiments to investigate the effect of shaped hole diameter.

Based on previous studies, the seed guide tube wears against seeds, which affects sowing quality (Kostić et al., 2018; Tang et al., 2024). In the flow field simulation analysis inside the seed guide tube, the goal was to allow maize seeds to have a relatively good falling velocity within the compo-site airflow and be carried by the airflow to reduce collisions with the inner wall of the tube. To achieve this, the flow deflector valve was first designed and analyzed. Using the intake configuration as the test factor, the gas flow pattern in the seed guide tube served as the test indicator. The air intake designs are categorized into three types: 0° air intake, 45° air intake, and annular diversion air intake. Next, after selecting the deflector valve type, the inner diameter of the seed guide tube was used as the test factor, and the steady-state airflow velocity inside the tube as the test indicator. The inlet pressure was set to 3 kPa, and single-factor experiments were conducted with the inner diameter of the seed guide tube set at four levels: 18 mm, 20 mm, 22 mm, and 24 mm.

##### 2.3.2.2 Coupled simulation test factors and indicators

###### 2.3.2.2.1 Single-factor experiment

The coupled simulation test model uses the optimal seed tray’s shaped hole diameter and seed guide tube inner diameter combinations based on the parameters above. Maize seeds fall from the sowing planter and enter the seed delivery tube at random positions. The working speed of the sowing planter is set to 29 r/min (simulating a sowing planter at 10 km/h). The experimental factor is the negative pressure in the seed tray, and the test indicator is the multiple seeding and miss seeding rate of maize seeds after they fall into the throw-seed tube. Each group of simulated test cycles lasts 10 s, is simulated three times, and is statistically analyzed.

###### 2.3.2.2.2 Multi-factor experiment

Conduct a two-factor, five-level, multi-factor experiment with positive pressure airflow in the seed guide tube. In preliminary research, single-factor experiments identified the optimal positive pressure range as 1–5 kPa. As shown in **Table 2**.

**Table 2:** Single-factor test factor level.

Horizontal	Experimental factors	
	Sowing planter negative pressure X_1_/(kPa)	Positive pressure in the seed guide tube X_2_/(kPa)
1	4.0	1.0
2	4.5	2.0
3	5.0	3.0
4	5.5	4.0
5	6.0	5.0

The coding settings for the factor levels in the multi-factor experiment are shown in **Table 3**.

**Table 3:** Test factor coding table.

Coding	Experimental factors	
	Sowing planter negative pressure X_1_/(kPa)	Positive pressure in the seed guide tube X_2_/(kPa)
1.414	6	5
1	5.707	4.414
0	5	3
-1	4.293	1.586
-1.414	4	1

### 2.4 Bench tests

#### 2.4.1 Test material

**Figure 6:**
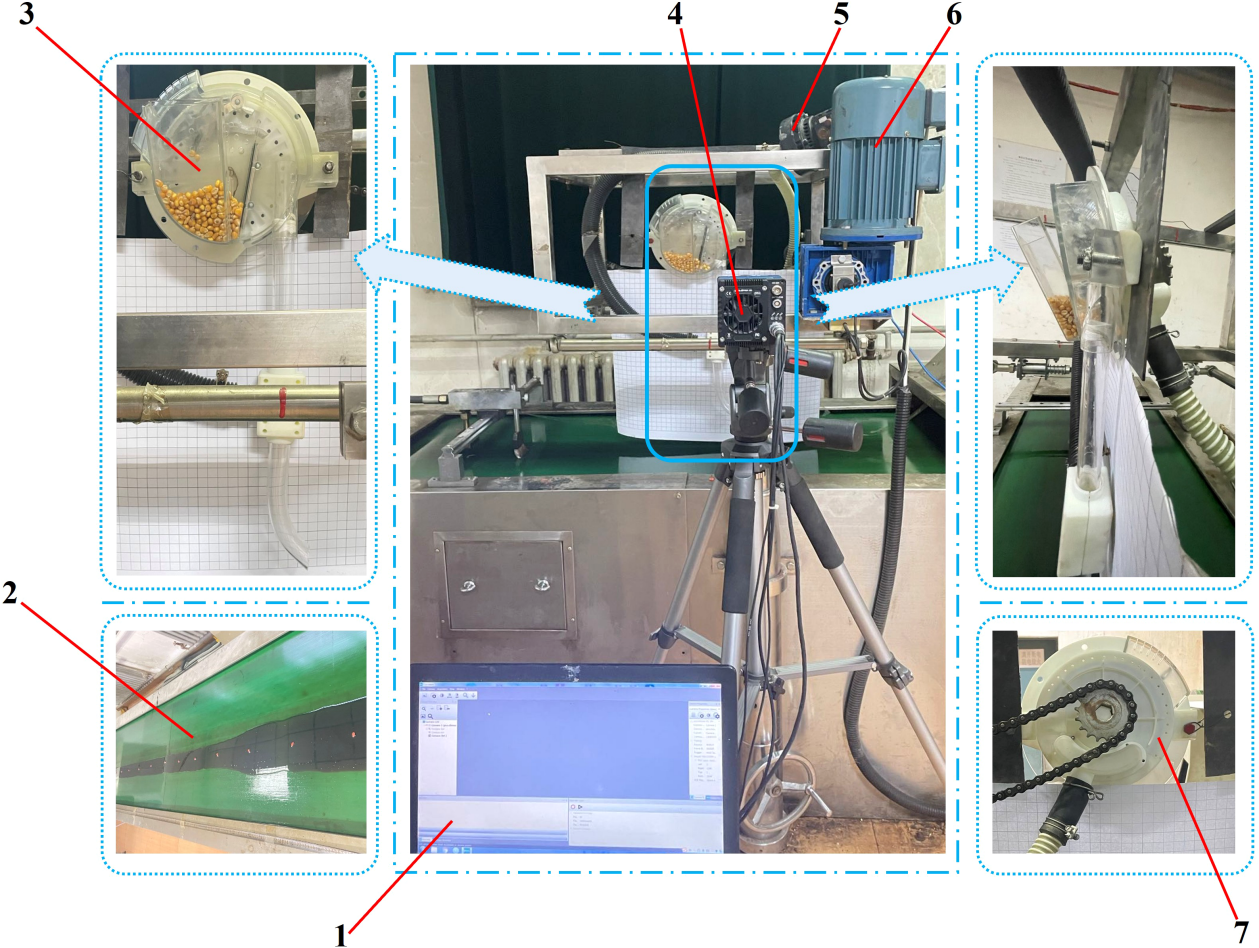
Seed sowing performance test bench. 1. data acquisition terminal 2. conveyor belt 3. pneumatic high-speed precision maize seed metering device 4. data acquisition camera 5. pressure-adjustable fan 6. drive motor 7. sprocket.

The selected test bench was the JPS-16 seed dispenser performance testing bench (Heilongjiang Provincial Agricultural Machinery Engineering Research Institute, Harbin, Heilongjiang). As shown in **Figure 6**.

#### 2.4.2 Test content and methods

Since it is impossible to accurately simulate the actual operating conditions of the sowing planter during testing, bench testing is necessary to verify the seeding performance. After selecting the stable airflow phase, the moment when the first maize seed leaves the throw-seed tube is set as zero. The time intervals between subsequent maize seeds leaving the throw-seed tube are then recorded. The spacing between seeds on the conveyor belt indicates multiple seeding or missed seeding. This test setup can automatically calculate the spacing between adjacent seeds and conduct statistical analysis on the data, comparing the effects of different negative and positive pressure levels on seeding quality.

According to the international standard ISO 7256/1, when the seed spacing exceeds 1.5 times the theoretical seed spacing, it is considered skipped seeding; when the seed spacing is less than 0.5 times the theoretical seed spacing, it is regarded as double seeding. In this study, the theoretical seed spacing was set at 220 mm. When the seed spacing exceeds 330 mm, it is classified as skipped seeding; when the seed spacing is less than 110 mm, it is classified as multiple seeding.

## 3 Results and discussion

### 3.1 Single-factor flow field analysis

#### 3.1.1 Simulation analysis of airflow field in sowing planter shaped hole

**Figure 7:**
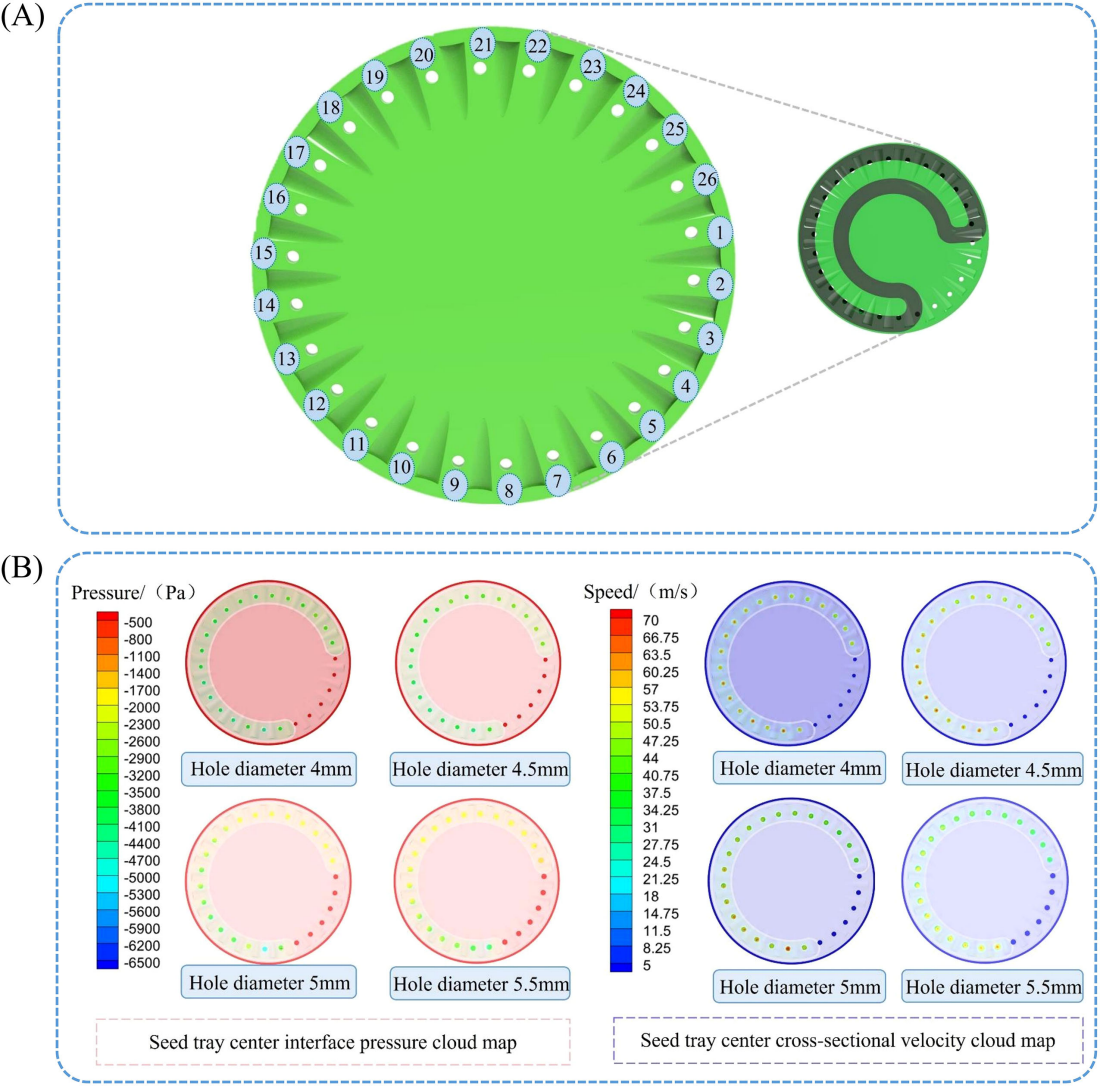
Seed tray shaped hole sequence and airflow field distribution in the tray center cross-section. **(A)** Seed tray’s shaped hole sequence; **(B)** Pressure and velocity cloud maps of the central section of the seed tray.

To facilitate observation of the airflow field simulation at various positions of the sowing planter shaped hole, the shaped holes are sorted in sequence as shown in **Figure 7A**.

**Figure 7B** below displays the post-processing results from the Fluent simulation of the flow field, showing the pressure and velocity contour plots of the center section of the seed tray.

As shown in the **Figure 7B**, when the negative pressure airflow enters, a certain amount of pressure loss occurs as it reaches the seed tray. The negative pressure on the shaped holes is greater at locations closer to the negative pressure inlet. However, as the shaped hole moves away from the inlet with the rotation of the seed tray, both the negative pressure and airflow velocity steadily decrease. For shaped holes numbered 1 to 6, the air pressure and velocity are substantially lower than those in other shaped holes within the negative pressure chamber. This is because the negative pressure chamber has excellent sealing properties, effectively preventing air leakage.

**Figure 8:**
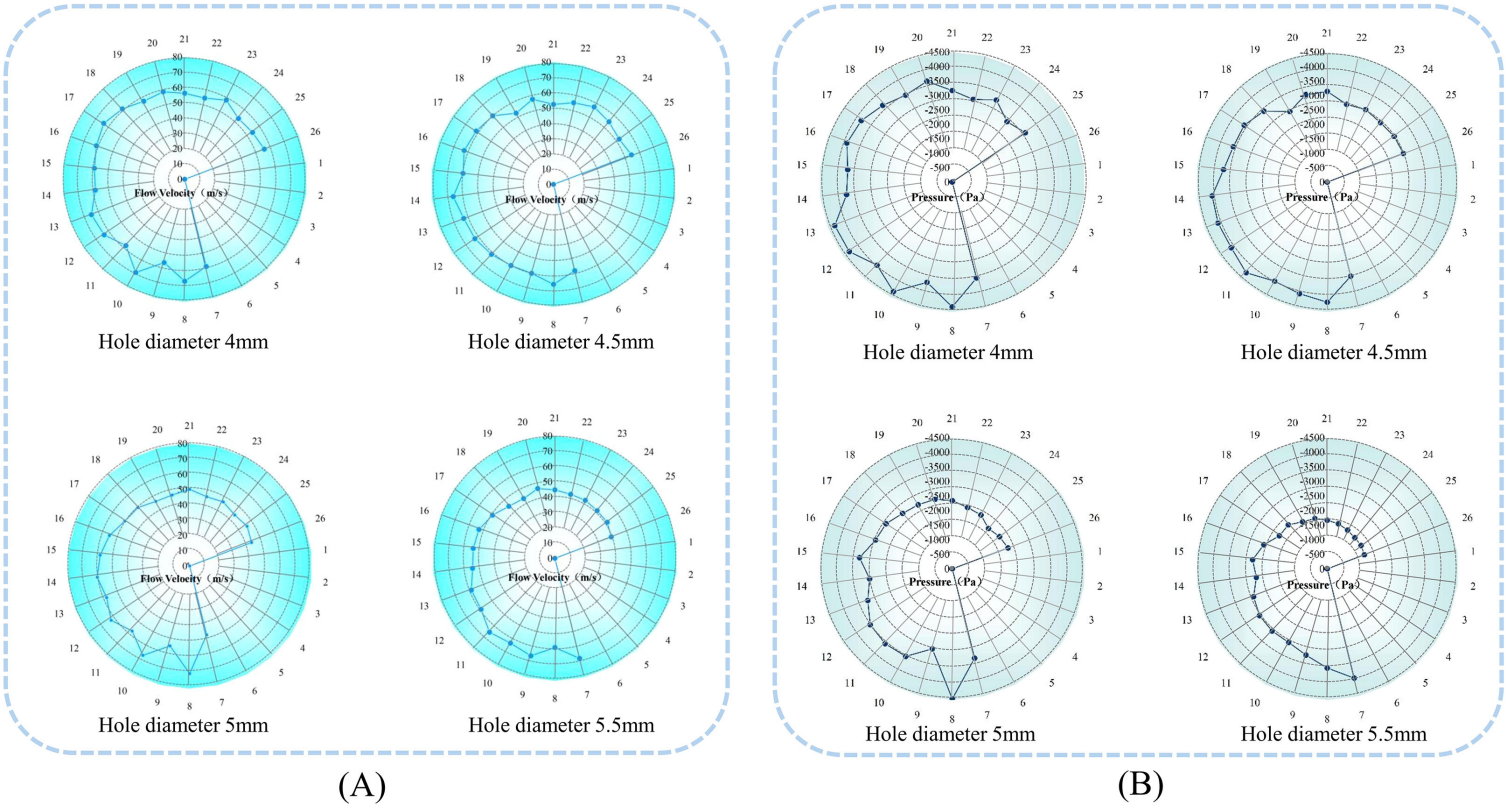
Effect of seed tray shaped hole diameter on internal airflow field characteristics. **(A)** Diagram of airflow velocity within the holes of different seeding plates; **(B)** Diagram of airflow pressure within the shaped holes of different seeding trays.

The stability of airflow velocity and pressure within the seed tray’s shaped hole is essential for ensuring seeds are smoothly adsorbed and transported to the seeding zone. Therefore, the airflow velocity and pressure within each shaped hole on the seed tray were extracted using post-processing software, and the data were imported into statistical analysis software for analysis. The average values and standard deviations of airflow velocity and pressure across different shaped hole diameters were calculated, and the data were visualized using Origin 2024 software, as shown in the **Figure 8**.

The analysis of **Figure 8A** shows that the diameter of the shaped hole in the seed tray is closely related to the stability of the airflow velocity inside the shaped hole. As the diameter of the shaped holes in the seed tray increases, the fluctuation of airflow velocity inside the shaped holes gradually intensifies. According to the data, the mean values and standard deviations of the airflow velocity in the four shaped holes are as follows: (59.62 ± 0.98) m/s, (58.76 ± 0.94) m/s, (53.29 ± 1.71) m/s, and (51.80 ± 2.02) m/s. Overall, as the diameter of the shaped holes in the seed tray increases, the airflow velocity inside the holes not only decreases but also becomes less stable.

Based on the analysis in **Figure 8B**, the increase in the shaped hole diameter of the seed tray is negatively related to the airflow pressure inside the shaped holes. Data shows that the average values and standard deviations of the air pressure inside the four shaped holes are as follows: (-3254.76 ± 358.03) Pa, (-3415.56 ± 118.46) Pa, (-2804.42 ± 147.88) Pa, and (-2273.96 ± 122.12) Pa. The analysis indicates that as the shaped hole diameter increases, the air pressure within decreases and becomes less stable. Further examination of the negative pressure zone shaped holes revealed that the average pressure was -3949.74 Pa when the hole diameter was 4 mm. In contrast, in the seed filling zone, shaped holes were created, and the average pressure decreased to -3139.65 Pa at a 5.5 mm diameter. These results demonstrate that increasing the shaped hole diameter of the seed tray results in greater air pressure loss inside the sowing planter. Additionally, smaller shaped hole sizes cause the pressure inside to be closer to the pressure at the air intake.

Analysis of air pressure distribution across different shaped hole: When the diameter of the seed tray’s shaped hole is 4 mm, the air pressure in most negative pressure zone holes is higher than -2700 Pa, but the pressure in shaped hole No. 9 suddenly drops to -3577.45 Pa, and the pressure in shaped hole No. 24 suddenly rises to 2798.63 Pa. Such drastic fluctuations in airflow pressure can easily cause seeds to detach during transportation due to sudden changes in adhesive force, leading to miss seeding.

When the shaped hole diameter increased to 5 mm and 5.5 mm, except for an abnormal surge in air pressure in shaped hole No. 8 of the 5 mm shaped hole seed tray, the pressure in the other shaped holes showed a steady downward trend in order of increasing number. However, the pressure in shaped holes 17 to 26 is lower than -2500 Pa, indicating severe air pressure loss in this area. The air pressure in some shaped holes is too low, requiring an increase in negative pressure at the air inlet, which will lead to increased energy consumption and violate the principle of low-power design.

Therefore, observing the seed tray with a shaped hole diameter of 4.5 mm showed that air pressure fluctuations were smallest in the critical migration shaped hole range of 7 to 26. The airflow inside the shaped hole creates a stable adhesive force to prevent seeds from falling out while avoiding extra energy use caused by low air pressure. As a result, the 4.5 mm diameter shaped holes in the seed tray can maintain a dynamic balance of airflow pressure during seed movement, effectively lowering the miss seeding rate while improving energy efficiency. This makes it the best choice for balancing seeding stability and energy saving.

#### 3.1.2 Simulation and analysis of flow field inside the seed guide tube

As a crucial component for accurate seed placement in the soil, the inner diameter of the air-assisted seed guide tube directly influences airflow capacity and seed movement. Variations in airflow velocity and pressure inside the tube determine not only the speed and stability of seed conveying but also whether seeds accumulate, clog, or collide with the tube walls during fall, leading to negative seeding phenomena. To enhance seed delivery efficiency and minimize these issues, a positive pressure assist seed guide tube is used to introduce airflow from the side inlet, increasing internal airflow velocity and pressure, which helps seeds move downward smoothly. However, if the tube diameter is too small, it can cause excessive airflow velocity, risking turbulence, backflow, and pressure fluctuations that may damage seeds or cause abnormal movements. Conversely, if the diameter is too large, it may lead to pressure loss and unstable airflow. Therefore, this study employs CFD-based single-factor simulation analysis to examine how the inner diameter of the seed guide tube affects internal pressure and airflow velocity, aiming to select the optimal structural parameters.

**Figure 9:**
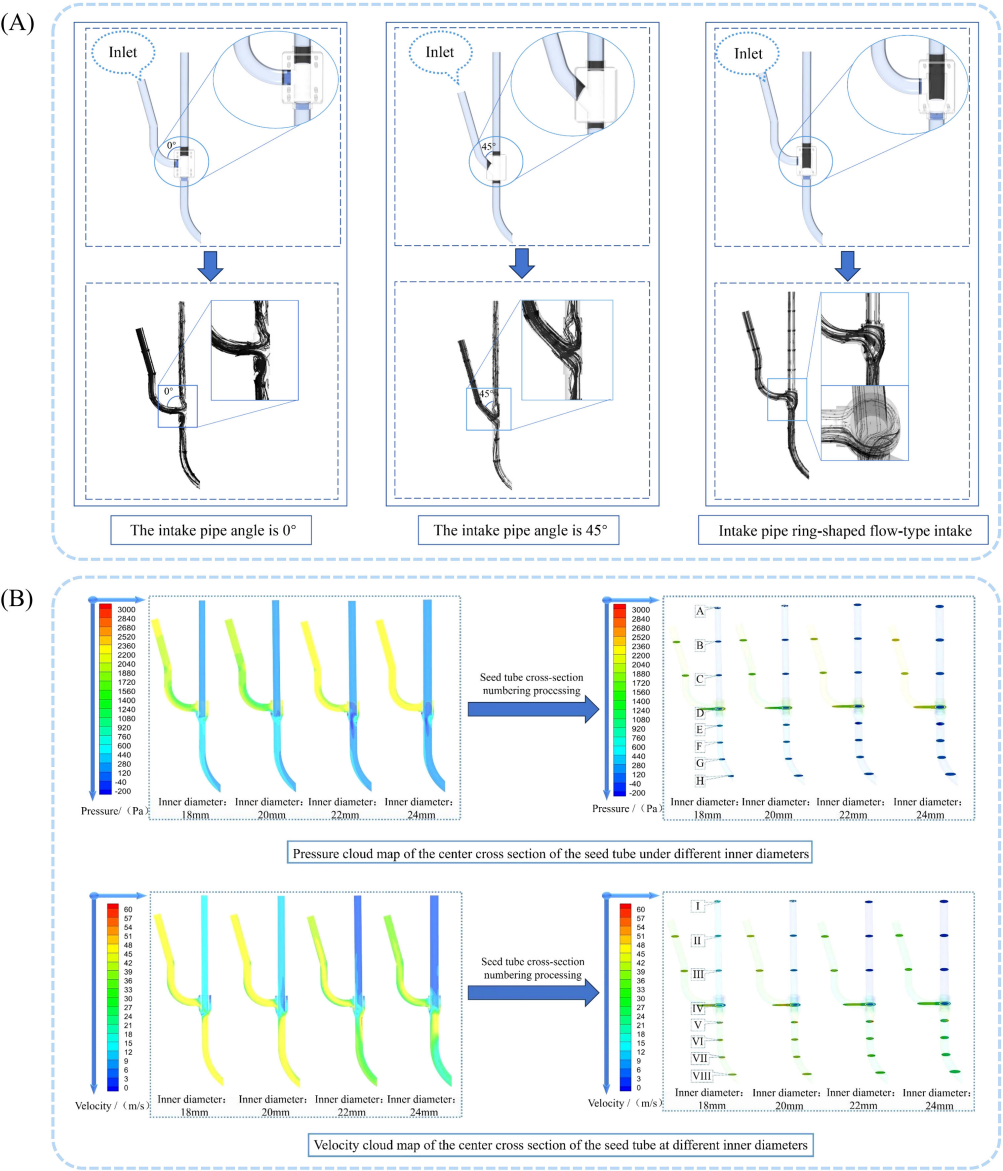
Effect of seed guide tube structural parameters on internal flow field characteristics. **(A)** Streamline diagram of gas flow within the under-constrained airflow-assisted seed guide tube; **(B)** Velocity and pressure contour maps at the center cross-section and cross-section of the seed guide tube under different seed guide tube inner diameter conditions.

Using the Fluent fluid simulation post-processing module to analyze the simulation results, we obtained the seed guide tube gas streamline diagrams for different intake configurations in the deflector valve, as shown in **Figure 9A**. Based on the diagram analysis, after the airflow passes through the intake tube, two main phenomena occur: first, a two-way diversion at the junction causes part of the airflow to flow upward along the tube, creating an upward branch airflow. This upward airflow pushes seeds during their fall, disrupting seed stability and reducing sowing quality. Second, under the influence of the ring-deflector valve, the airflow enters the throw-seed tube, significantly accelerating the seeds within the tube. Additionally, the seeds are less likely to collide with the inner walls because of the enveloping effect of the ring-shaped airflow, which helps achieve optimal seed delivery. Therefore, the ring-shaped airflow inlet configuration is chosen.

Use the Fluent fluid simulation post-processing module to analyze the simulation results and generate the airflow velocity and pressure contour maps at the center cross-section and transverse cross-section of the seed guide tube. To better understand the pressure and velocity of the airflow within the seed guide tube, different cross-sections were positioned at specific points. Three cross-sections were taken at intervals of 100 mm along the center of the seed guide tube, one at the flow deflector valve, and four at intervals of 50 mm along the throw-seed tube. The codes A, B, C, D, E, F, G, and H represent the airflow pressure inside the seed guide tube, while I, II, III, IV, V, VI, VII, and VIII represent the airflow velocity inside the tube. Due to the high-speed airflow in the inlet, a downward negative pressure is generated inside the seed guide tube, applying a downward force on the seeds. Similarly, high-speed airflow in the throw-seed tube also creates a downward force. For analytical purposes, the pressure exerting a downward force on the seeds inside the seed guide tube is considered positive, consistent with that in the throw-seed tube. The airflow results are displayed in **Figure 9B**.

**Figure 10:**
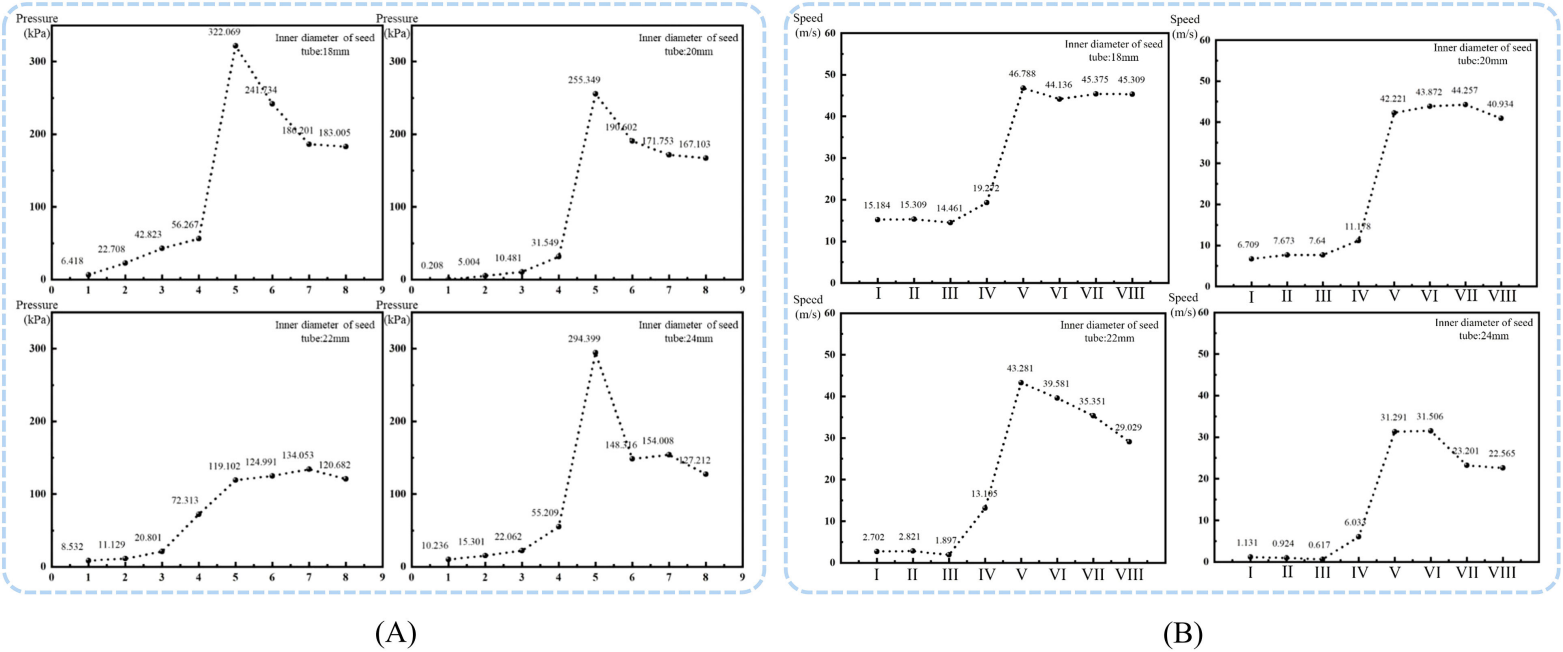
Airflow field distribution at specific cross-sections inside the seed guide tube. **(A)** Air pressure in sections A to H of the seed guide tube; **(B)** Airflow velocity in sections I to VIII of the seed guide tube.

For easier analysis, the Fluent simulation data was exported and processed using Tecplot 360 EX 2021 software (Tecplot, USA). The pressure at sections numbered A to H and I to VIII was selected, and the effect of the seed guide tube diameter on air pressure and flow velocity was determined, as shown in **Figure 10**.

Analysis shows that in different inner diameter seed guide tubes, both air pressure and flow velocity first increase and then decrease, with maximum values at cross-section E and cross-section V at the deflector valve inlet. Cross-sections F-H and VI-VIII are located in the bent tube section, which obstructs airflow and causes a decrease in air pressure and flow velocity. When the seed guide tube’s inner diameter is 18 mm, 20 mm, or 24 mm, the air pressure remains high with strong acceleration capacity, but the fluctuation amplitude is large and stability is poor, making uniform seed acceleration difficult. Conversely, the 22 mm inner diameter seed guide tube has a smoothly changing air pressure without sudden shifts. Although 18 mm and 20 mm seed guide tubes have higher flow rates, their small diameter can cause seeds, with an average length of 12.35 mm, to collide violently, affecting sowing quality. The airflow velocity changes in 22 mm and 24 mm tubes are similar. Considering both flow rate and pressure, the optimal inner diameter for the seed guide tube was determined to be 22 mm.

### 3.2 CFD-DEM results and discussion of CFD-DEM coupled simulation

#### 3.2.1 Single-factor experiment

This study examines the seed transport efficiency during the seed filling phase of the sowing planter. It aims to evaluate the seed filling performance of a pneumatic high-speed precision maize seed metering device and provide a reference for subsequent full-machine prototyping and bench testing. Using negative pressure as the experimental variable and miss seeding rate as the indicator, to reduce simulation time, the number of leaked seeds was counted after the seed tray rotated ten times. The negative pressure airflow was set between 1 and 6 kPa, the seed tray speed was maintained at 29 r/min, and the positive pressure airflow in the seed guide tube was set to 3 kPa. All other parameters remained constant, and a single-factor experiment was conducted to assess the impact of negative pressure airflow.

**Figure 11:**
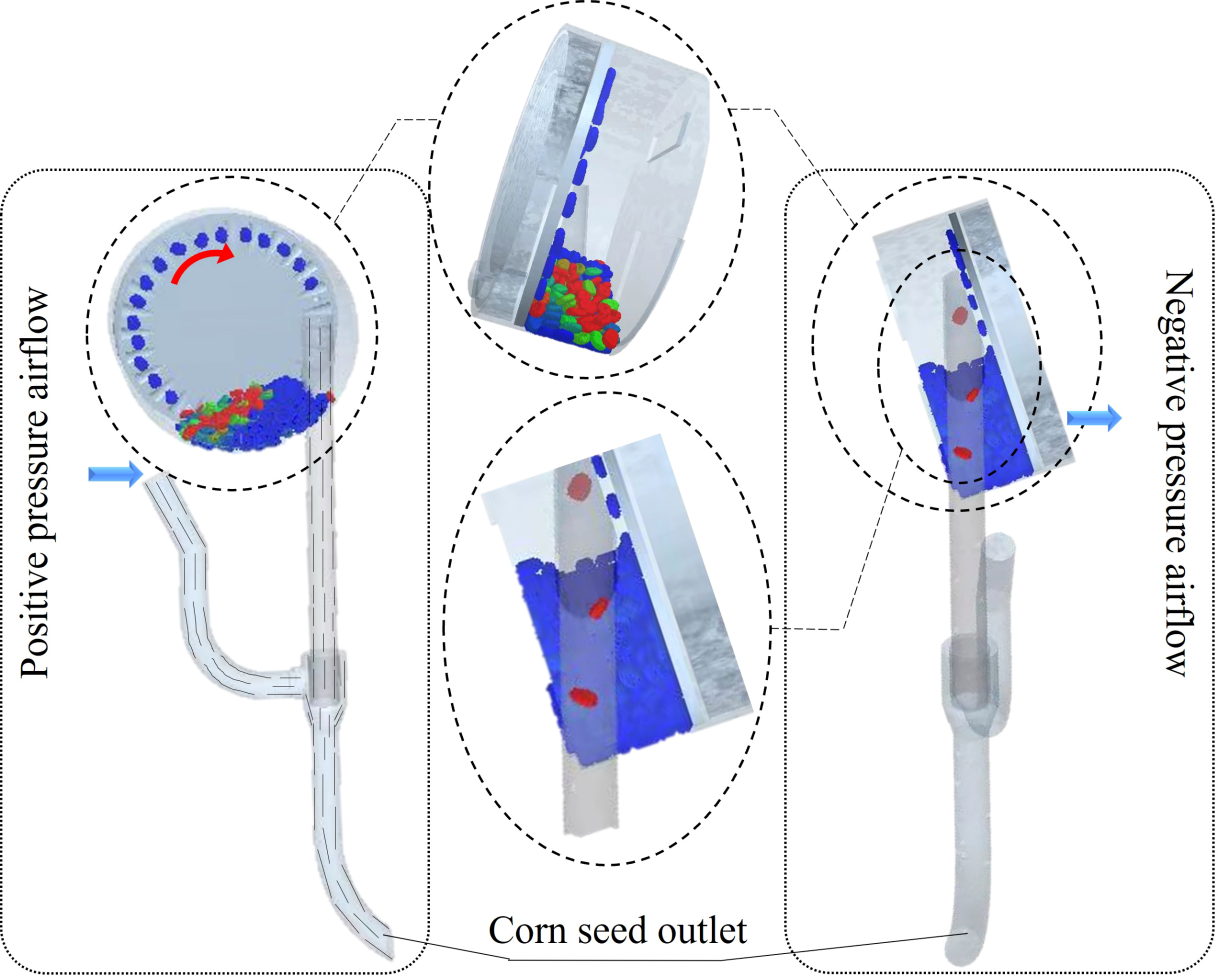
Seed metering system coupling simulation process.

As shown in **Figure 11**, the seeding system can operate smoothly at a working speed of 29 r/min. The corrugated seed tray and negative pressure airflow effectively disturb the seed population, allowing the seeds to successfully adhere to the seed tray and fall smoothly into the seed guide tube to complete the seed distribution process. Process the coupled simulation results and count the number of missed seedings during ten rotations of the seed tray after airflow stabilization. The test results are presented as mean ± standard deviation.

**Figure 12:**
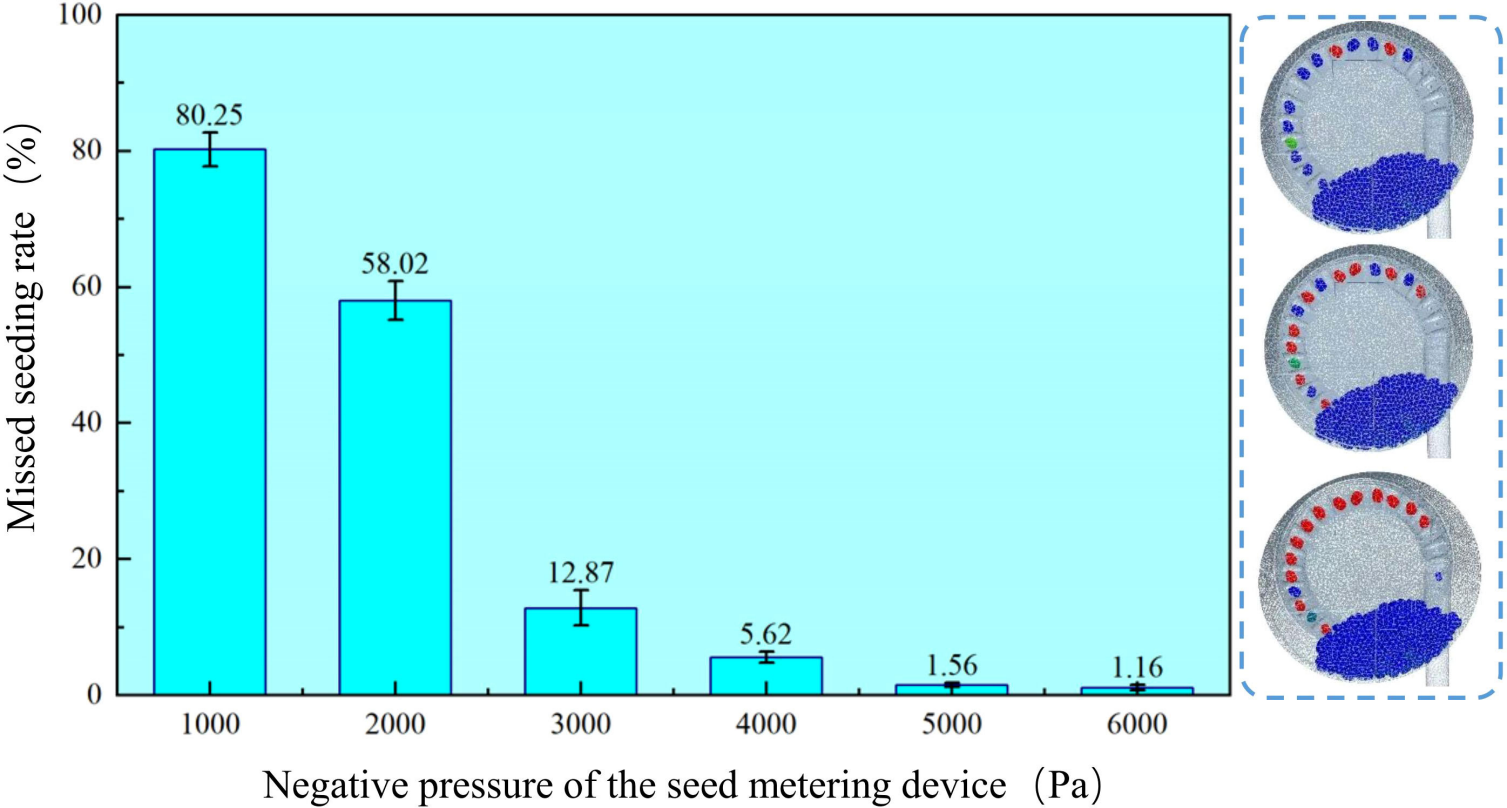
Effect of negative pressure on the miss seeding rate.

According to the data in the **Figure 12**, when the negative pressure is -1 kPa, the miss seeding rate is highest at 80.25%, while at -6 kPa, the miss seeding rate is lowest at 0.16%. It is clear that as the negative pressure of the sowing planter increases, the miss seeding rate continues to decrease. The miss seeding rate is much higher at -1 kPa and -2 kPa than at -3 kPa to -6 kPa. This suggests that when the negative pressure of the sowing planter is too low, the seed tray cannot effectively adsorb the maize seeds. As the seed tray rotates, the maize seeds fall back into the seed population, causing miss seeding. The miss seeding rate at -3 kPa is higher than at -4 to -6 kPa. Therefore, subsequent multi-factor tests will be conducted at -4 to -6 kPa, combined with positive pressure in the seed guide tube, to identify the optimal parameter combination.

#### 3.2.2 Multi-factor experiments

In the single-factor experiment above, the negative pressure range that guarantees seeding performance was identified. To make the experiment more practical, we added positive pressure airflow to the seed guide tube. We performed a two-factor, five-level orthogonal experiment to find the best parameter combination. The results are shown in **Table 4**.

**Table 4:** Test design scheme and results.

Serial number	Experimental factors		Performance indicators	
	Negative pressure A/(km/h)	Positive pressure B/(kPa)	Qualification index Q/(%)	Coefficient of variation C/(%)
1	-1	-1	93.34	11.30
2	1	-1	90.19	16.05
3	-1	1	98.65	13.71
4	1	1	93.57	15.56
5	-1.414	0	95.33	9.94
6	1.414	0	89.79	16.37
7	0	-1.414	91.05	14.05
8	0	1.414	96.86	15.03
9	0	0	94.25	11.43
10	0	0	94.81	11.19
11	0	0	94.28	11.38
12	0	0	94.21	11.88
13	0	0	94.15	11.32
14	0	0	94.88	11.96
15	0	0	94.64	11.31
16	0	0	94.88	11.43

The test results obtained in **Table 4** were used to analyze the effects of the factors on the qualification index and the coefficient of variation of the seeding performance indicators.

##### 3.2.2.1 The impact of various factors on the qualification index

The qualification index is a key parameter for assessing seed selection performance (Zhao et al., 2022; Sun et al., 2025). Statistical analysis of the experimental data was carried out using Design-Expert 10.0.1 software (Stat-Ease, Inc., Minneapolis, MN, USA), including an analysis of variance (ANOVA) on the experimental factors affecting the qualification index. This analysis examined the significance of each factor and its interactions. The results of the variance analysis for each factor and interaction are shown in **Table 5**. The software develops a mathematical model of how the influence factors affect the performance test index, the qualification index, and the regression equation is shown in Equation 10:

**Table 5:** Variance analysis of each factor on the qualification index.

Sources of variance	Sum of squared deviations	Degree of freedom	F-value	P-value (significance)
regression model	74.21	5	87.67	<0.0001 (***)
factor *x*_1_	32.26	1	190.54	<0.0001 (***)
factor *x*_2_	35.73	1	211.03	<0.0001 (***)
factor *x*_1_^2^	5.20	1	30.71	0.0002 (***)
factor *x*_2_^2^	3.39	1	5.23	0.4720 (N)
factor *x*_1_ *x*_2_	0.095	1	0.56	0.0410 (**)
error	0.72	7		
total	75.91	15		

Note: In the analysis of variance process, P < 0.01 (highly significant, ***), P < 0.05 (significant, **), P < 0.1 (moderately significant, *), and N is not significant.


(10) $$y_{1}=94.51-2.01x_{1}+2.11x_{2}-0.48x_{1}x_{2}-0.81x_{1}^{2}-0.11x_{2}^{2}$$

where *y*_1_ is the coded value of the seed spacing qualification index, with units of %; *x*_1_ is the coded value of the negative pressure of the sowing planter, with units of kPa; *x*_2_ is the coded value of the positive pressure of the seed guide tube, with units of kPa.

As shown in **Table 5**, the analysis of variance indicates that factors *x*_1_, *x*_2_, *x*_1_^2^, and *x*_1_ *x*_2_ are significant terms in the regression model. Among these, the significance levels of factors *x*_1_, *x*_2_, and *x*_1_^2^ are less than 0.01, indicating that these factors have an extremely significant impact on the qualification index. The significance level of factor *x*_1_ *x*_2_ is less than 0.05, suggesting that it has a significant effect on the qualification index. The regression equation is meaningful, and the residual term is not significant, which shows that the regression model fits well.

**Figure 13:**
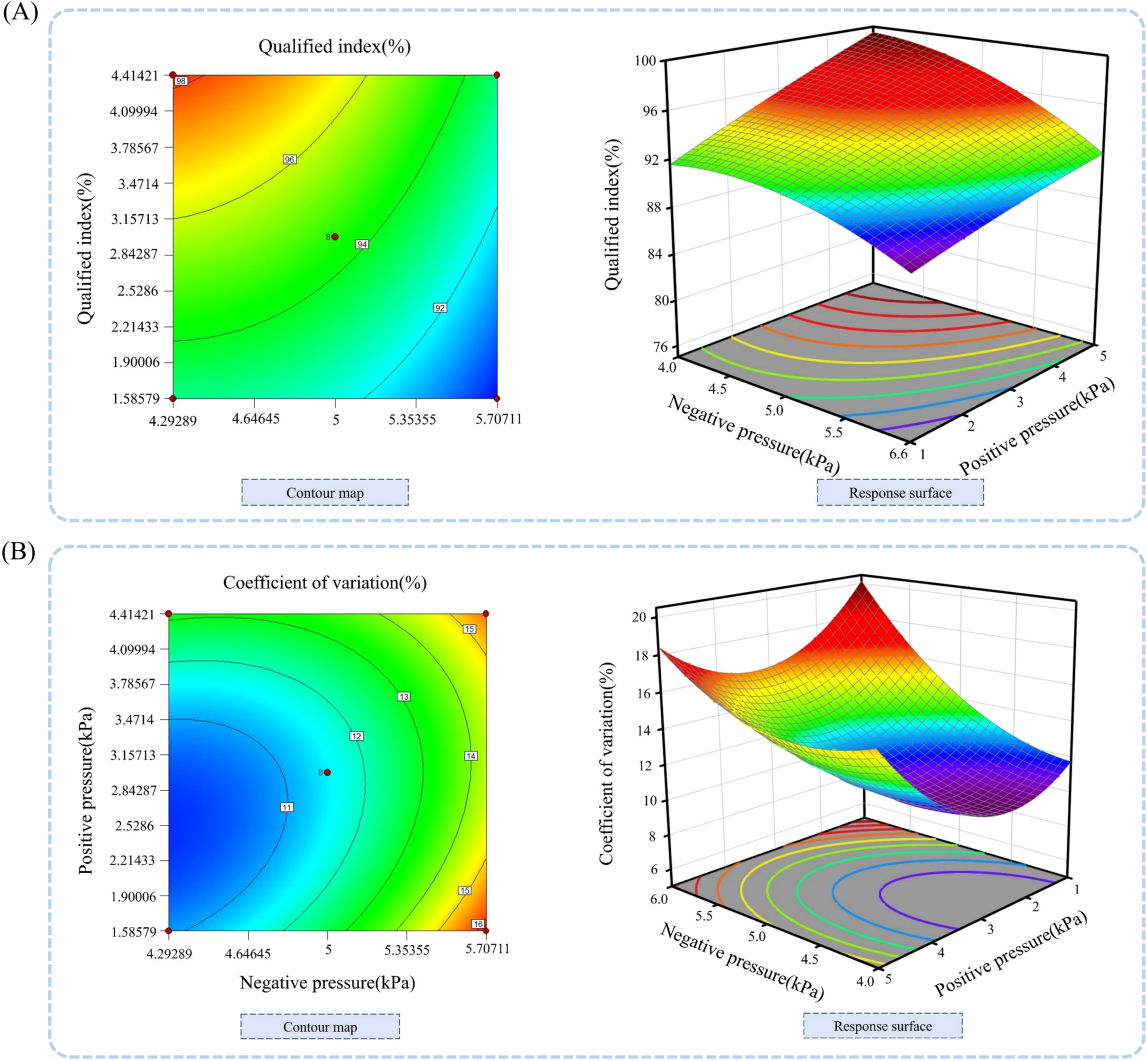
Response surface analysis of the effects of positive and negative pressure on seed dispensing performance indicators. **(A)** Contour plots and response surfaces for each factor on the qualification index; **(B)** Contour plots and response surfaces for each factor on the coefficient of variation.

To demonstrate how different factors affect the qualification index of performance indicators, Design-Expert 10.0.1 software was used to generate response surface diagrams showing the impact of negative pressure in the sowing planter and positive pressure in the seed guide tube on the qualification index, as shown in **Figure 13A**.

By analyzing the response surface plot in **Figure 13A**, we can assess the effects of various factors and their interactions. When the negative pressure remains constant, the qualification index increases as the working pressure rises; when the positive pressure is held steady, the qualification index decreases as the negative pressure increases. The analysis shows that the qualification index varies significantly with changes in forward velocity. Based on the results of the variance analysis, the factors influencing the qualification index of the seeding performance are, in order of importance from highest to lowest, negative pressure and positive pressure.

##### 3.2.2.2 Analysis of the influence of various factors on the coefficient of variation of seed sowing performance indicators

Statistical analysis of the experimental data was carried out using Design-Expert 10.0.1 software. An analysis of variance was performed on the experimental factors affecting the coefficient of variation to assess the significance of each factor and interaction. The results of the variance analysis for each factor and interaction are presented in **Table 6**. The software develops a mathematical model of how the influence factors impact the performance test index, specifically the qualification index, and its regression equation is and its regression equation is shown in Equation 11.

**Table 6:** Variance analysis of each factor to the coefficient of variation.

Sources of variance	Sum of squared deviations	Degree of freedom	F-value	P-value (significance)
regression model	61.45	5	79.52	<0.0001 (***)
factor *x*_1_	30.79	1	199.20	<0.0001 (***)
factor *x*_2_	1.37	1	8.84	0.0140 (**)
factor *x*_1_^2^	6.63	1	42.93	<0.0001 (***)
factor *x*_2_^2^	20.56	1	133.04	<0.0001 (***)
factor *x*_1_ *x*_2_	2.10	1	13.60	0.0042 (***)
error	0.54	7		
total	62.99	15		

In the analysis of variance process, P < 0.01 (highly significant, ***), P < 0.05 (significant, **), P < 0.1 (moderately significant, *), and N is not significant.


(11) $$y_{2}=11.49+1.96x_{1}+0.41x_{2}-0.73x_{1}x_{2}+0.91x_{1}^{2}+1.60x_{2}^{2}$$

where *y*_2_ is the coded value of the seed spacing coefficient of variation, with units of %; *x*_1_ is the coded value of the negative pressure of the sowing planter, with units of kPa; *x*_2_ is the coded value of the positive pressure of the seed guide tube, with units of kPa.

From the analysis of variance (ANOVA) of the coefficient of variation for each factor and their interactions in **Table 6**, it is clear that five factors—*x*_1_, *x*_2_, *x*_1_^2^, *x*_2_^2^, and *x*_1_ *x*_2_—were identified as significant terms in the regression model. Among these, the significance levels of factors *x*_1_, *x*_1_^2^, *x*_2_^2^, and *x*_1_ *x*_2_ were less than 0.01, indicating that these factors have an extremely significant impact on the coefficient of variation. Factor *x*_1_ *x*_2_ has a significance level below 0.05, indicating a significant effect on the coefficient of variation. The regression equation is meaningful, and the term for model deviation is not significant, suggesting that the regression model fits the data well.

To clearly show how various factors influence the coefficient of variation of performance indicators, Design-Expert 10.0.1 software was used to generate response surface plots illustrating the effects of sowing planter forward velocity and working pressure on the coefficient of variation, as shown in **Figure 13B**.

Analyzing the response surface plot and contour plot in **Figure 13B** allows us to examine the effects of different factors and their interactions. When the negative pressure of the sowing planter remains constant, the coefficient of variation initially decreases and then increases as the positive pressure of the seed guide tube rises. Conversely, when the positive pressure stays steady, the coefficient of variation gradually increases with higher negative pressure. The analysis shows that the coefficient of variation changes notably when the negative pressure of the seed dispenser varies. Combined with the variance analysis results, the factors affecting the coefficient of variation of seed dispensing performance indicators, ranked from most to least significant, are negative pressure, positive pressure, and negative pressure of the seed guide tube.

#### 3.2.3 Experimental optimization

To find the best combination of working parameters for the sowing planter to achieve optimal seeding performance, a multi-objective variable optimization method is used to determine the ideal parameters. The experimental factor range is set as the boundary conditions, and a nonlinear programming parameter equation is established as shown in Equation 12:


(12) $$\left\{ \begin{array}{l} \max y_{1}\\ \min y_{2}\\ \text{s.t}.\;4\text{kPa}\leq x_{1}\leq 6\text{kPa}\\ \;1\text{kPa}\leq x_{2}\leq 5\text{kPa}\\ \;0\leq y_{1}\leq 1\\ \;0\leq y_{2}\leq 1 \end{array}\right.$$

Using the Design-Expert 10.0.1 software to optimize and determine each parameter, the optimal operating settings for the sowing planter were found to be a negative pressure of -4.209 kPa, rounded to -4 kPa; the working pressure is -3.395 kPa, rounded to -3 kPa; the qualification index is 96.48%; and the coefficient of variation is 10.90%. The seeding performance is optimal and can be confirmed in subsequent bench tests.

#### 3.2.4 Exploration of the influence law of seed guide performance

By analyzing the results of the coupled simulation, it can be observed that the seeds fall smoothly into the seed guide tube after dropping from the seed tray. The maize seeds in the seed guide tube can accelerate smoothly within the tube. Therefore, this validates the conclusion from the previous experiments that an inner diameter of 22 mm for the seed guide tube meets the seeding requirements of the pneumatic high-speed precision maize seed metering device.

**Figure 14:**
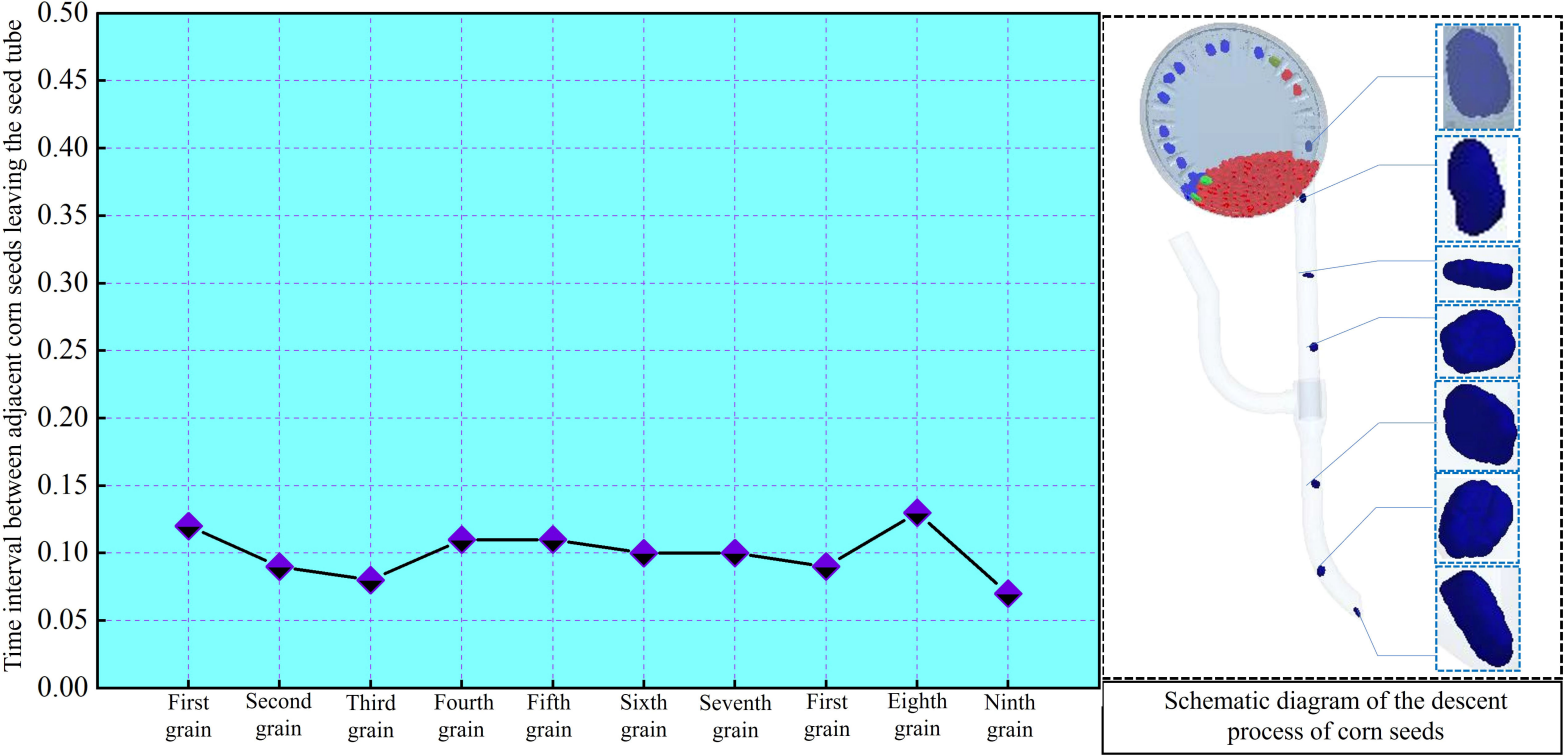
Interval time of maize grains leaving the end of the throw-seed tube in different postures.

Record the time interval between each seed’s fall and the previous seed, then visualize the data as shown in **Figure 14**.

The average value and standard deviation of the time interval between seed releases from the seed guide tube are calculated as (0.08 ± 0.013) seconds. It is clear that different gestures of maize seeds have minimal impact on seed guide timing, and seed spacing remains fairly consistent. Therefore, the uniformity of seed throwing from the throw-seed tube is satisfactory.

The under-constrained airflow-assisted seed guide tube designed in this study for air-assisted throw seed has a certain restrictive effect on the movement trajectory of maize seeds after they leave the sowing planter. By enveloping the maize seeds with airflow, it helps guide their descent. This reduces friction between the maize seeds and the tube wall. Additionally, airflow assistance decreases collisions between the seeds and the tube wall. In the coupled simulation, only a few collisions between the maize seeds and the inner wall of the seed guide tube are observed. Therefore, the under-constrained airflow-assisted seed guide tube in this study can significantly lower the coefficient of variation in seed spacing.

### 3.3 Bench validation test results and analysis

To verify the validity of the optimal parameter combination selected for the coupled simulation, sowing performance tests were conducted in bench tests. The simulation shows that the average time interval between seeds leaving the end of the throw-seed tube is 0.08 s, with a standard deviation of 0.013 s. In this bench test, the forward velocity of the seed metering devices is reflected in the speed of the conveyor belt. Based on preliminary research by the team, the best seeding performance was achieved at a forward velocity of 10 km/h, so the forward velocity was set to 10 km/h. Based on the forward speed combined with the time intervals derived from the above simulation, the simulated average seeding grain spacing was calculated to be (222 ± 36.14) mm, as shown in Equation 13:


(13) $$L=vt_{z}$$

where *L* is the distance between adjacent maize seeds, with units of m; *v* is the forward speed of the seed metering devices, with units of m/s; *t_z_* is the time interval between adjacent maize seeds leaving the throw-seed tube, with units of s.

**Figure 15:**
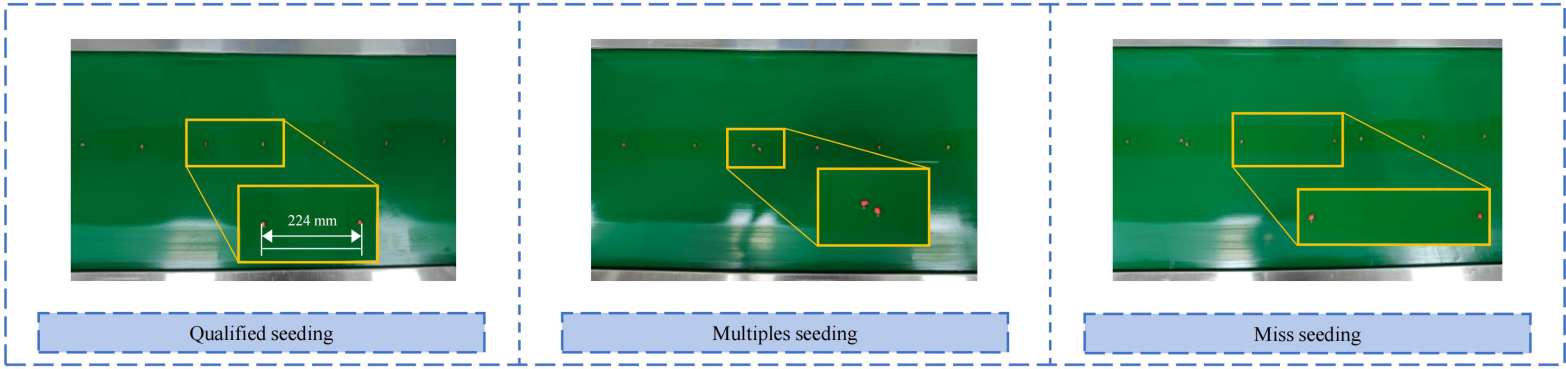
Seed distribution diagram after completion of the seed metering device operation.

In the bench test, wait for the airflow to stabilize, then record the seed distribution on the conveyor belt, as shown in **Figure 15**. The actual seed spacing distribution was compared with the theoretical seed spacing calculated in the previous stage: the theoretical average seed spacing was 222 mm, calculated from the simulation of the seeding time interval and forward speed, while the actual average seed spacing was 224 mm, and the theoretical seeding grain spacing was 220 mm in the results of the bench test. Although occasional multiple seeding and miss seeding occurred in the bench test, the two showed highly consistent performance in terms of seeding consistency, with an error of less than 5%, further verifying the accuracy of the prediction results from the coupled CFD-DEM simulation and the practicability of the optimal parameter combinations.

Therefore, bench testing not only verified that the structural parameters and air pressure combination design proposed in the simulation have great practical value, but also confirmed from an experimental perspective that the pneumatic high-speed precision maize seed metering device offers excellent stability and uniformity under high working conditions and holds promising prospects for promotion and application.

### 3.4 Discussion

In this study, we systematically investigated the effects of key structural parameters, such as the shaped hole diameter of the seed tray and the inner diameter of the seed guide tube, on seeding performance in a pneumatic high-speed precision maize seed metering device through CFD single-factor and CFD-DEM coupled simulations. Subsequently, multi-factor experiments were conducted, and the optimal parameter combination was determined by matching negative and positive pressures. Under this setup, the time intervals between adjacent seed drops were recorded in the simulation, and the theoretical seed spacing was calculated. Finally, the actual seed spacing was compared through bench tests, and the results from the simulation and tests showed high consistency, indicating that the optimized structural parameters proposed in this paper are feasible and practical under optimal operating conditions.

Domestic and foreign research on pneumatic high-speed maize precision seeding systems has accumulated relatively rich results. Mechanical seed metering devices in high-speed operations often miss seeding, result in multiple seeding, and cause seed injury and other problems. Therefore, in recent years, domestic and foreign experts have gradually turned their attention to the study of pneumatic seed metering devices. Bustos-Gaytán et al. (2025) carried out a systematic analysis of the airflow transport parameters of a pneumatic seed metering device, pointing out that there is a nonlinear coupling between the airflow field and the seed attitude, and that consistency needs to be ensured by fine parameter tuning. He et al. (2024) and Wang et al. (2022) explored the mechanisms of different air cavity structures and the role of seed flow fields using the CFD-DEM method, but primarily focused on low-velocity and medium-velocity conditions. Domestically, Zhou et al. (2024) proposed the design of an annular air-blowing assisted seed guide tube, but showed the problem of uneven airflow and high seed collision rate at high speeds; Tang et al. (2024) designed a high-speed precision dual-chamber maize seed metering device that emphasized the seed attitude adsorption characteristics, but the study on the interaction law between the negative and positive pressures was not sufficiently investigated.

Compared with the above studies, the present study is located in the high-speed working condition (10 km/h), while most of the existing studies focus on the low and medium speeds; secondly, the present study not only investigates the negative pressure adsorption process alone, but also reveals the significant interaction between the negative pressure and the positive pressure through the CFD-DEM simulation and bench test, which is not yet sufficient in the previous multifactorial optimization studies of pneumatic seed metering devices. This paper further refines how sowing hole diameter influences local air pressure and velocity. It shows that a shaped hole diameter of 4.5 mm can sustain a high average adsorption force and effectively reduce air pressure fluctuations caused by interference between holes, thus achieving a dynamic balance of air pressure distribution within a porous system. This finding surpasses the previous single hypothesis of “smaller hole diameter adsorbing more stably” and establishes a better balance between adsorption stability and energy use. Additionally, aligning with the conclusion by El-Emam et al. (2021) that “hole diameter and adsorption force exhibit a nonlinear relationship”, this study also finds that under high-speed conditions, overly large hole diameter can cause the pressure fluctuation of the edge shaped hole in the negative pressure chamber to rise by over 20%. This phenomenon is often ignored in low-speed research.

In terms of seed guide tube structure optimization, this study suggests 22 mm as the optimal inner diameter, ensuring that the airflow has enough drag force to steadily push the maize seeds downward while preventing violent turbulence and seed rebound caused by excessive flow velocity. This study employed a circular deflector valve design to enhance the uniformity of the airflow velocity field in a 22 mm diameter tube by 40% and cut the collision frequency between seeds and the tube wall by 60%, significantly boosting seed spacing consistency during high-speed transport and addressing the performance shortcomings of traditional straight seed guide tubes under high-speed conditions. Compared with the 20 mm design proposed by Wu et al. (2020), the improved airflow distribution in this paper creates a more continuous enveloping flow field below the deflector valve, enhancing seed distribution uniformity.

Further analysis showed that both the negative pressure in the sowing planter and the positive pressure in the seed guide tube significantly influence seeding consistency, and their interaction is also significant, indicating that the system needs coordinated design of pneumatic parameters to optimize the entire transport process. This study has some limitations: first, the experiment was only conducted on the “DeMeiya No. 1” maize variety, and seed size may limit the generalizability of the results. In the future, it will be necessary to develop relevant models for seeds of different grain types; second, the bench test did not account for environmental factors such as soil resistance and vibration in the field. In real-world operation, airflow disturbances at the end of the seed guide tube could impact seeding accuracy, and further field tests are necessary. Third, the simulation model did not include the effect of seed surface roughness on adhesion force. Future work could improve the model by incorporating a microscopic contact model based on the discrete element method.

## 4 Conclusion

This study addresses common issues such as miss seeding and multiple seeding that often occur during high-speed precision maize planting. A pneumatic high-speed precision maize seed metering device was designed, and CFD and CFD-DEM simulations along with bench tests were conducted on its key structural parameters. The following conclusions were drawn:

1. Analysis of seed tray shaped hole diameter: After a comprehensive review of airflow intensity and adsorption stability, the best shaped hole diameter was found to be 4.5 mm. This size results in minimal airflow fluctuations and strong stability, ensuring high single-seed adsorption efficiency while effectively reducing miss seeding rates and energy use.
2. Analysis of the inner diameter of the seed guide tube: The airflow in the seed guide tube creates pressure and velocity peaks near the inlet. An inner diameter that is too large or too small can lead to airflow instability, sudden changes in velocity, or seed collisions. Simulation and bench test results consistently show that a seed guide tube with an inner diameter of 22 mm offers the best airflow stability and seed delivery consistency.
3. Coupled simulation and bench test verification: The seeding performance was confirmed through CFD-DEM simulation and bench tests. Under conditions of -4 kPa negative pressure on the seed tray, 3 kPa positive pressure on the seed guide tube, and a forward velocity of 10 km/h, the seed seeding qualification index reached a maximum of 96.48%, and the coefficient of variation was minimized at 10.90%. This meets the requirements for high-speed precision seeding with good uniformity.
4. This study provides information about the innovative design of a high-speed precision sowing planter and advocates for the development of pneumatic seeding methods. It serves as a reference for exploring seeding approaches for different types of seeds. Future research can thoroughly consider the effects of soil resistance, sowing planter vibration, and seed roughness on seeding performance and carry out additional experimental studies.

## Data Availability

The original contributions presented in the study are included in the article/supplementary material. Further inquiries can be directed to the corresponding authors.
